# Colocalization of Different Influenza Viral RNA Segments in the Cytoplasm before Viral Budding as Shown by Single-molecule Sensitivity FISH Analysis

**DOI:** 10.1371/journal.ppat.1003358

**Published:** 2013-05-09

**Authors:** Yi-ying Chou, Nicholas S. Heaton, Qinshan Gao, Peter Palese, Robert Singer, Timothée Lionnet

**Affiliations:** 1 Department of Microbiology, Icahn School of Medicine at Mount Sinai, New York, New York, United States of America; 2 Department of Medicine, Icahn School of Medicine at Mount Sinai, New York, New York, United States of America; 3 Department of Anatomy and Structural Biology, Albert Einstein College of Medicine, Bronx, New York, New York, United States of America; 4 Gruss Lipper Biophotonics Center, Albert Einstein College of Medicine, Bronx, New York, New York, United States of America; Harvard Medical School, United States of America

## Abstract

The Influenza A virus genome consists of eight negative sense, single-stranded RNA segments. Although it has been established that most virus particles contain a single copy of each of the eight viral RNAs, the packaging selection mechanism remains poorly understood. Influenza viral RNAs are synthesized in the nucleus, exported into the cytoplasm and travel to the plasma membrane where viral budding and genome packaging occurs. Due to the difficulties in analyzing associated vRNPs while preserving information about their positions within the cell, it has remained unclear how and where during cellular trafficking the viral RNAs of different segments encounter each other. Using a multicolor single-molecule sensitivity fluorescence *in situ* hybridization (smFISH) approach, we have quantitatively monitored the colocalization of pairs of influenza viral RNAs in infected cells. We found that upon infection, the viral RNAs from the incoming particles travel together until they reach the nucleus. The viral RNAs were then detected in distinct locations in the nucleus; they are then exported individually and initially remain separated in the cytoplasm. At later time points, the different viral RNA segments gather together in the cytoplasm in a microtubule independent manner. Viral RNAs of different identities colocalize at a high frequency when they are associated with Rab11 positive vesicles, suggesting that Rab11 positive organelles may facilitate the association of different viral RNAs. Using engineered influenza viruses lacking the expression of HA or M2 protein, we showed that these viral proteins are not essential for the colocalization of two different viral RNAs in the cytoplasm. In sum, our smFISH results reveal that the viral RNAs travel together in the cytoplasm before their arrival at the plasma membrane budding sites. This newly characterized step of the genome packaging process demonstrates the precise spatiotemporal regulation of the infection cycle.

## Author Summary

Influenza A viruses cause one of the major respiratory infection diseases in humans. The viruses possess a genome consists of eight different RNA segments and the incorporation of all the eight RNA segments is required for the generation of an infectious virus particle. The precise process of how these eight viral RNA segments are co-packaged into progeny virus particles remains undefined due to the limitations of methodology to determine the locations of different vRNA segments in infected cells with single-molecule resolution. In this study, we established an experimental system to examine the localization of different viral RNA segments in an infected cell with high spatial precision. We found that viral RNA belonging to different segments gather together in the cytoplasm which is facilitated by cellular recycling endosomal protein Rab11. Our results supported the idea that eight different viral RNAs likely form a super-complex as they travel to the site for virion incorporation. These findings extend our knowledge on the process of influenza virus genome packaging and suggest a mechanism by which the genome assembly of different viral RNA segments is regulated.

## Introduction

The Influenza A virus genome consists of eight negative-sense, single-stranded RNAs. In a virus particle or an infected cell, the viral RNAs exist in the form of viral ribonucleoprotein complexes (vRNPs) with the viral RNA (vRNA) encapsidated by the nucleoproteins (NP) and associate with the polymerase complex [1] . Since each vRNA encodes one to two essential viral proteins, the packaging of all eight vRNAs is required for the production of an infectious virus particle. Multiple pieces of evidence have shown that influenza virus selectively packages eight different vRNPs into virus particles [2], [3], [4], [5], [6]; however, when and where the selection occurs during viral infection remains unknown. The separation and assembly of different vRNA segments is difficult to determine, due to the limitation of methodology applicable to detect different vRNA segments with single-molecule sensitivity and preserve their spatial distributions in the infected cells. In this report, we established a single-molecule sensitivity fluorescence *in situ* hybridization (smFISH) system which detects and localizes influenza vRNAs in an infected cell, allowing the determination of the spatial relationship among different vRNPs. The assembly process of different vRNA segments during virus infection can therefore be studied with high resolution. Influenza virus is one of the rare RNA viruses which has a nuclear replication phase [1]. The vRNPs first have to be released from the virion by the disassociation of matrix protein and then they are imported into the nucleus for viral mRNA transcription and vRNA synthesis. The nuclear import of vRNPs is an active process that involves the cellular import machinery. Nuclear localization signals on the NP protein are recognized by importin α (karyopherin α) and together the vRNP and importin α form a tri-complex with importin-β that is actively transported into the nucleus through the nuclear pore complex [7]. It has been shown with biochemical analysis that the replicating vRNPs were associated with densely packed chromatin while the newly synthesized vRNPs are released into the nucleoplasm [8]. In addition, it was observed that the NP proteins were distributed to the apical face of the nucleus when the export of vRNPs was inhibited [9]. This suggested that the polarized transport of vRNPs started when they were in the nucleus. The newly assembled vRNPs are exported into the cytoplasm, where they are targeted to the plasma membrane for packaging. The export of vRNPs was also dependent on the cellular export machinery and the influenza nuclear export protein (NEP) [10], [11]. It has been demonstrated that leptomycin B treatment efficiently abrogated the export of vRNPs, showing that the transport of vRNPs out of the nucleus is a Crm1 dependent pathway [10], [12]. Even though the mechanism of vRNPs transport into and out of the nucleus has been elucidated, it is unknown whether different segments of the incoming vRNPs enter the nucleus separately or as a package and whether the newly synthesized vRNPs gather into a super-complex in the nucleus to be exported.

It has been suggested that the reassortment of influenza vRNAs happens in the cytoplasm because when the cytoplasm of two different virus infected cells was fused, the segments from the two virus strains were incorporated into progeny viruses in a random manner [13]. It is thus possible that the selection of vRNPs to be packaged happens after nuclear export. The trafficking route of the exported vRNPs to the apical plasma membrane has been shown to involve the cellular cytoskeletal system [14] and Rab11-positive recycling endosomes [15], [16], [17]. Live-cell imaging using an antibody specifically for vRNP showed that the complex moves along microtubules rapidly in both directions. Depolymerization of microtubules using nocodazole was shown to disrupt the apical targeting of the nucleoproteins, implicating the microtubule network in the polarized transport of vRNPs [16]. Rab11 is a small GTPase regulatory protein that has been shown to be localized to the endocytic recycling endosomes and plays an essential role in regulating recycling to the plasma membrane [18]. Several studies have shown that apical transport of influenza vRNPs depends on the interactions between Rab11 and the polymerase protein PB2 [17], [19]. It was unknown whether different vRNA segments traffick together with the Rab11 recycling endosomes to the plasma membrane or whether the colocalization and sorting of different vRNAs to be packaged happens at the plasma membrane.

In this present study, we applied single-molecule sensitivity fluorescence *in situ* hybridization assay to visualize vRNAs of different segments in influenza virus infected cells. By using this technique, the degree of colocalization between two different vRNAs can be determined with great spatial precision. By performing smFISH and colocalization analysis on virus infected cells at different time points post infection, we found that the incoming vRNPs remain associated until they are imported into the nucleus. Newly synthesized vRNPs were detected at different locations in the nucleus and the newly exported vRNPs of different identities gather together at later stages of replication when the vRNPs are loaded onto Rab11 positive vesicles. Herein, we have provided evidence that progeny vRNPs of different identities travel together in the cytoplasm. These results likely suggest that different vRNPs are selected into a pre-formed super-complex during their trafficking to the plasma membrane, where budding and genome packaging occur.

## Results

### Single-molecule sensitivity FISH and colocalization analyses of influenza viral RNAs in infected cells

To better understand the interaction pattern between vRNAs of different identities in influenza virus infected cells, we established a single-molecule sensitivity fluorescence *in situ* hybridization (smFISH) system for influenza vRNAs to determine their locations in infected cells. Single-molecule sensitivity was obtained using 48 single-fluorophore-labeled short DNA oligos targeting different regions of the same vRNA. The targeted vRNAs were bound by multiple probes at the same time giving high fluorescence intensity signals, allowing them to be distinguished as diffraction-limited spots. The number of spots and the location of their centers in 3-dimensional space can then be determined using - imaging processing programs. When 48 Cy5 labeled probes against the PB2 segment were used, fluorescence spots can be observed in influenza A/Puerto Rico/8/34 (PR8) virus infected cells at 4 hours post infection (hpi) but no spots were detected in the mock infected cells (Fig. S1A). This demonstrated that the smFISH system was highly specific for vRNAs. The fact that the fluorescence spots detected by the imaging analysis program appear homogenous in size as diffraction-limited spots as well as the fact that the fluorescent particles display a unique, well-defined fluorescence intensity peak are strong indications that single molecules of vRNAs were detected using this system (Fig. S1B). To further test the specificity of the probe sets against different species of influenza vRNAs, probe sets targeting the HA vRNA encoding the head regions of the H1 subtype and the H9 subtype were designed and synthesized. The probes for the H1 HA vRNA were labeled with Cy3 fluorophores and those for the H9 HA vRNA were labeled with Cy5 fluorophores. The two differently labeled probe sets were mixed and used for *in situ* hybridization on cells infected with wild type PR8 virus harboring the H1 HA segment or with the recombinant PR8 cH9/1 virus which contains an HA segment coding the head region from an H9 subtype HA and a stalk from an H1 HA. In cells infected with the PR8 virus, fluorescent spots can only be observed in the Cy3 channel but not from the Cy5 channel. This demonstrated the specific binding of probes against the H1 HA vRNAs. On the other hand, fluorescent spots can only be detected in the Cy5 channel in cells infected with the recombinant PR8 cH9/1 viruses, showing the specific hybridization of the probes against the H9 HA vRNAs (Fig. S1C). This experiment demonstrated the high sequence specificity of the smFISH system and showed that the specificity could be preserved when two probe sets targeting different species of vRNAs were mixed during the hybridization process. This allowed the detection of vRNAs of different segments in the same cell at the same time.

**Figure 1:**
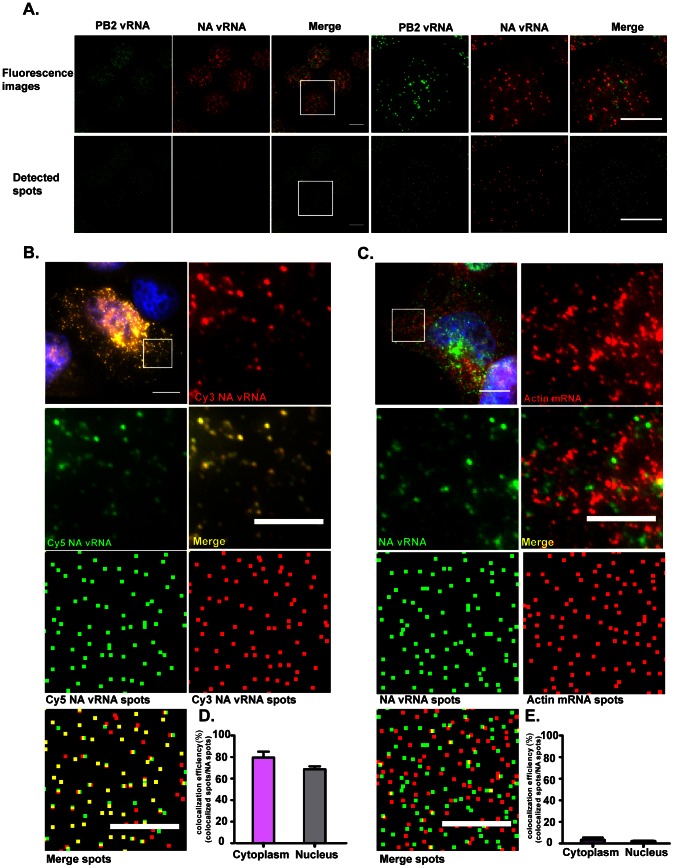
Single molecule sensitivity FISH and colocalization analyses of influenza viral RNAs. (**A**) Images of MDCK cells infected with PR8 at MOI = 5 and fixed at 2 hpi (Maximum Intensity projections). The cells were probed against PB2 and NA vRNA using Cy5 labeled probes against the PB2 segment and Cy3 labeled probes against the NA segment. The upper panel shows the maximum projection of the fluorescent image stacks acquired in the Cy5 and Cy3 channels. The lower panel shows the 2D representation of the detected spots corresponding to the fluorescent images above. Each is represented as a square. Magnified images of the squared regions are shown on the right of each panel. The merge images of the detected spots show the spatial relationship between the Cy5 and Cy3 spots. Scale bar = 10 µm. (**B**) MDCK cells were infected with PR8 virus at MOI = 5 and fixed at 6 hpi (Maximum Intensity Projection). Two probe sets, 24 Cy3 labeled probes and 24 Cy5 labeled probes targeting different regions of the NA vRNA were used for hybridization. Top left: merged fluorescent image of DAPI, Cy3 and Cy5 channels is shown (scale bar = 10 µm). The magnified boxed area in each color channel and the corresponding detected spots are shown for comparison. Scale bar = 5 µm. (**C**) Colocalization analysis of influenza NA vRNA and cellular β-actin mRNA. MDCK cells were infected with PR8 virus at MOI = 5 and fixed at 8 hpi (Maximum Intensity Projection). The cells were hybridized with Cy5 labeled probes against the NA vRNAs, and Cy3 labeled probes against the host β-actin mRNA. The magnified boxed area in each color channel and the corresponding detected spots are shown for comparison. Scale bar = 10 µm in the low magnification image and 5 µm in the magnified images. (**D**) Colocalization efficiency of the Cy3 and Cy5 spots corresponding to the NA vRNA is shown. The colocalization efficiency is determined as the ratio of colocalized spots to NA spots. (**E**) Quantification of the colocalization efficiency of influenza NA vRNA and β-actin mRNA at 8 hpi. The colocalization efficiency is calculated by dividing the number of colocalized spots by the number of NA vRNA spots.

**Figure 2:**
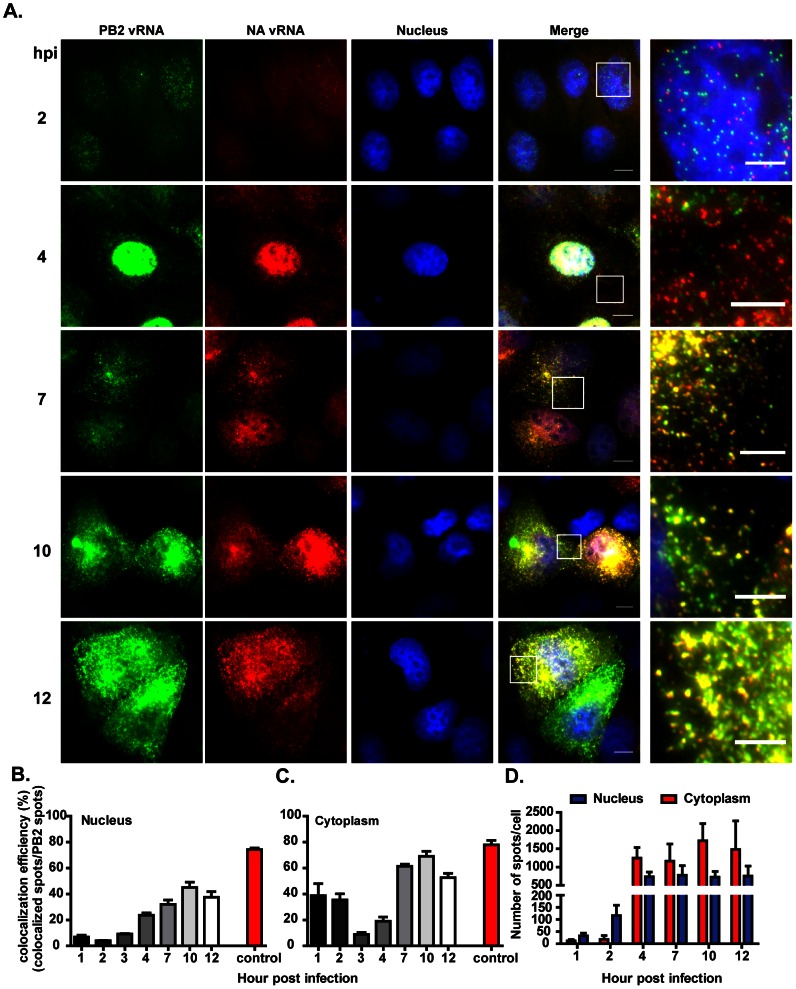
Colocalization of the PB2 and NA vRNAs in PR8 virus infected cells at different time points post infection. MDCK cells were infected with PR8 virus at MOI = 5 and fixed at different time points post infection. Two-color FISH was then performed using Cy5 labeled probes against the PB2 vRNAs and Cy3 labeled probes against the NA vRNAs. (**A**) Fluorescent images representing the PB2 vRNA (Cy5), NA vRNA (Cy3) and nuclei (DAPI). Typical images taken at 2, 4, 7, 10 and 12 hpi are presented in each lane (Maximum Intensity Projections). Scale bar = 10 µm. Zoomed-in views of the boxed regions are shown in the rightmost panel of each lane. Scale bar = 5 µm. Image Contrast was adjusted to make cytoplasmic single molecules apparent. Due to their high vRNA density, some nuclei appear uniformly bright with these settings. (**B**) **and** (**C**) Quantification of the colocalization efficiency between PB2 and NA vRNAs in the nucleus (**B**) and the cytoplasm (**C**). The control colocalization efficiency represents the efficiency detected between Cy3 and Cy5 signals when the two differently labeled probe sets are both targeting the NA vRNA. (**D**) Average copy number per cell of PB2 and NA vRNAs in infected cells at different time points post infection. The average vRNA copy number per cell is calculated by dividing the sum of PB2 and NA vRNA molecules detected by the number of nuclei in the image. A total of five images containing more than 80 cells were analyzed for each time point.

In order to measure the degree of colocalization between vRNAs of different identities, cells infected with viruses were fixed and hybridized using two sets of probes targeting two different vRNAs, one labeled with Cy3 and the other labeled with Cy5. The center of each spot corresponding to a vRNA molecule was then located in 3-D space using the custom spot detection algorithm. Mapping of the different colored spots revealed the distances among spots and colocalization efficiency between the two vRNA segments could then be determined (Fig. 1A). A custom designed colocalization analysis tool was developed to measure the distances between color spots and their nearest neighbor spots of a different color. This allows the quantification of the number of spots being colocalized in individual cells. To validate the system, two control experiments were performed. The positive control experiment was done using two differently labeled probe sets (Cy3 and Cy5) targeting different regions of the same vRNA. Figure 1B showed the images of NA vRNAs detected by two differently labeled probe sets. The high degree of colocalization between the Cy3 and Cy5 spots in cells infected with PR8 virus at 6 hpi demonstrated the specificity and the sensitivity of our colocalization analysis. Quantitative colocalization analysis of the images showed that 80% of the Cy3 and Cy5 spots colocalized in the cytoplasm and 70% of them colocalized in the nucleus (Fig. 1D). To exclude the possibility that the high colocalization efficiency detected could be due to high density of vRNAs, we tested the colocalization efficiency between the highly expressed cellular β-actin mRNA and NA vRNA. The MDCK cells infected with PR8 virus were hybridized with Cy3 labeled probes for β -actin mRNA and Cy5 labeled probes targeting the NA vRNA. At 8 hpi, we observed NA vRNAs in the nucleus and the cytoplasm, whereas the β -actin mRNAs were mainly in the cytoplasm. The copy number of β-actin mRNA detected in the cytoplasm was 1409±283, similar to what was detected for viral RNAs at 4–8 hour post infection (Fig. 2D). When merging the two channels, a very small degree of colocalization between the two RNA species was observed (Fig. 1C). Quantification of the colocalization between the beta-actin mRNAs and NA vRNAs also showed a low percentage of colocalization, approximately 2.5% in the nucleus and 5% in the cytoplasm (Fig. 1E). These results demonstrated the specificity and sensitivity of our quantitative colocalization analysis.

### PB2 and NA viral RNAs colocalized in the cytoplasm after 4 hour post infection

To investigate the colocalization kinetics of different viral RNA species during the influenza virus life cycle, we performed smFISH and colocalization analysis on two vRNAs: PB2 and NA at different time points post infection. Figure 2A shows the typical images of the distributions of PB2 and NA vRNAs at different hour-post infections. At 2 hpi, the PB2 and NA vRNA molecules were observed mainly within the nucleus with only a few in the cytoplasm. New vRNAs were synthesized at this time point; however, the PB2 and NA vRNAs were observed in different locations in the nucleus. No colocalization between the PB2 and NA vRNAs was observed (Fig. 2B). Even though few spots were detected in the cytoplasm, the PB2 and NA vRNAs present high colocalization efficiency in this compartment. It is likely that the colocalized spots represent vRNAs of incoming virus particles which have not yet entered the nucleus. As the vRNAs were being replicated, the nucleus became packed with newly synthesized vRNAs at 4 hpi. It should be noted that the measured colocalization efficiency between PB2 and NA vRNAs increased over time in the nucleus, albeit reaching significantly lower level compared to the control. The extreme accumulation of vRNAs in the nucleus at later time points resulted in a very high spatial density of fluorescence spots. In this concentration regime, actual physical interactions between single PB2 and NA vRNAs became difficult to distinguish from crowding-induced random proximity. This effect could account for the higher colocalization fraction measured in the nucleus at later time points using our assay (Fig. 2B). The smFISH system also allowed the quantification of the copy number of vRNAs in individual cells. By doing quantification of copy number of vRNAs in the nucleus and cytoplasm compartments in each cell, it was observed that increased vRNAs can be detected in the nuclei at 2 hpi, demonstrating that the replication of vRNA started as early as 1 hour post infection. Nuclear export of newly synthesized vRNAs likely occurred after 2 hpi because higher copy number of vRNAs could be detected in the cytoplasm at 4 hpi as compared to that at 2 hpi (Fig. 2D). In fact, the newly replicated vRNAs could be seen in the cytoplasm at 3 hpi (Fig. S3A) and these vRNAs were distributed throughout the entire cytoplasm. When the colocalization between the PB2 and NA vRNAs was analyzed at 4 hpi, low colocalization efficiency was observed (Fig. 2C). This suggested that the vRNAs were exported out of the nucleus individually and vRNAs of different segments were not traveling together at the early phase post export. At later stages, large number of vRNAs was observed in the cytoplasm. Accumulation of vRNAs at the peri-nuclear regions was observed around 6–7 hpi, which is consistent with previous reports [17] (Fig. S3B). At this time point, 60% of the NA vRNAs colocalized with the PB2 vRNAs (Fig. 2C). The colocalized spots were not only detected at the peri-nuclear regions where the vRNA accumulated but also spread out in the cytoplasm. These results suggested that the PB2 and NA vRNAs started to gather and travel together between 6–7 hpi (Fig. 2&S3). As infection progressed, more vRNAs were seen in the cytoplasm and higher colocalization efficiency between the PB2 and NA vRNAs was detected. At 10 and 12 hours post infection, a significant increase of the released virus particles was observed (Fig. S4) and the colocalized vRNAs were detected mainly in the cytoplasm and near the apical surface of the cells, which likely represent the vRNAs being packaged into the budding virions. The quantification of colocalization between the PB2 and NA vRNAs in the nucleus and cytoplasm at different hours post infection is shown in Figure 2B and 2C. In order to test whether the timing of association was specific to the PB2 and NA vRNAs or a feature common to all viral segments, we measured the colocalization of five other pairs of viral RNAs. All pairs shared similar kinetics with that for the PB2 and NA vRNAs, demonstrating that the temporal pattern of association is general (Fig. S5).

### Incoming viral RNAs travel together before nuclear import

**Figure 3:**
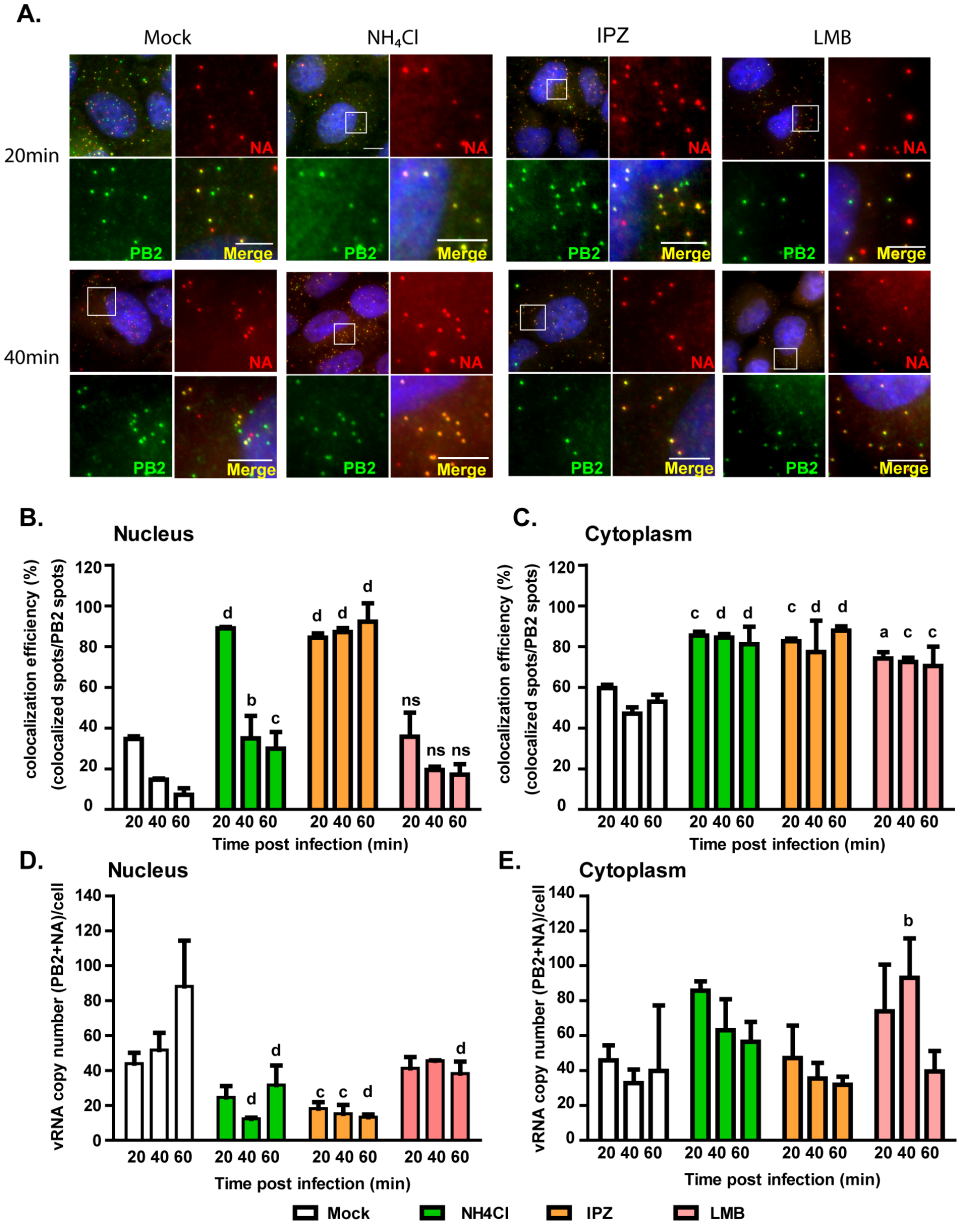
Colocalization of PB2 and NA vRNAs during the first hour of infection. MDCK cells were incubated in medium containing DMSO, 20 mM ammonium chloride (NH_4_Cl), 100 µM importazole (IPZ) or 40 ng/ml leptomycin B (LMB) during PR8 virus infection at MOI = 100. Two-color FISH was performed at 20 min, 40 min and 60 min post infection with Cy5 probes targeting the PB2 vRNA and Cy3 probes targeting the NA vRNAs. (**A**) For each experimental condition, four Maximum Intensity Projections of the 3D fluorescent images are shown. The upper left image shows the merged image of the Cy5, Cy3 and DAPI channels. DAPI staining (blue) stains the nucleus of the cell. The enlarged images of the square area are shown on the upper right (NA, red spots), the lower left (PB2, green spots) and the lower right (merged image of the Cy3 and Cy5 signals with DAPI). Scale bar = 5 µm. (**B**) Colocalization efficiency of PB2 and NA in the nuclei of PR8 virus infected MDCK cells treated with different drugs at 20 min, 40 min and 60 min post infection. (**C**) Colocalization efficiency of PB2 and NA in the cytoplasm of PR8 virus infected MDCK cells treated with different drugs at 20, 40 and 60 min post infection. The average copy number of vRNAs (sum of PB2 and NA vRNAs) in the nucleus (**D**) and the cytoplasm (**E**) of cells treated with different drugs are shown. For each experimental condition more than 40 cells were analyzed. An unpaired student t-test was performed for each time point between experimental groups with the mock treated group. a: p value<0.05, b: p value<0.01, c: p value<0.001, d: p value<0.0001, ns: no significance.

Influenza virus particles enter the cells through endocytosis. Acidification of the endosomes allows the viral envelope to fuse with the endosomal membrane; influx of protons into the virus particles releases the vRNPs from the matrix protein, freeing the vRNPs into the cytosol [1]. It is unclear whether the released viral RNAs stay together or travel individually to the nucleus. In order to understand this process in more detail, MDCK cells were infected with PR8 virus at MOI = 100 and the cells were treated with ammonium chloride (NH_4_Cl), leptomycin B (LMB) or importazole (IPZ), to disrupt different steps of influenza vRNP nuclear transport. The colocalization efficiency of PB2 and NA vRNAs was then analyzed at 20, 40 and 60 minutes post-infection. In the mock treated cells, both PB2 and NA vRNAs could be detected in the nucleus as early as 20 minutes post infection (Fig. 3A). Replication of vRNAs started around 40 minutes post infection because the average copy number of vRNAs detected in the nucleus were comparable at 20 and 40 minutes post infection, while a significant increase was detected at 60 minutes post infection (Fig. 3D). When the colocalization of PB2 and NA vRNAs were assessed during the first hour post infection, colocalization of vRNPs in the nucleus decreased over time (from approximately 30% to 5%) while colocalization of vRNPs remained around 50% in the cytoplasm (Fig. 3B&C). This shows that vRNPs imported into the nucleus became separated, and suggests that they replicated at different positions in the nucleus. While some PB2 and NA vRNAs were not colocalized in the cytoplasm at early time points post infection, it is possible that the released vRNPs depart from each other and travel individually to the nucleus or the non-colocalized vRNPs are molecules that shuttle back into the cytoplasm after nuclear import. First, to demonstrate that the colocalized PB2 and NA vRNPs detected in the cytoplasm in the early time points post infection were predominantly vRNPs from the same virion, we retained the vRNPs in the entering virions by treating the cells with 20 mM of ammonium chloride. Ammonium chloride increases the pH value in the endosomal compartments, preventing the release of vRNPs from the matrix proteins; therefore, the vRNPs were kept in the incoming virus particles. When the cells were treated with 20 mM ammonium chloride, most of the PB2 and NA vRNAs were colocalized in the cytoplasm throughout the first hour of infection (Fig. 3A); the colocalization efficiency between these two vRNA species was also significantly higher than that in the mock treated cells (Fig. 3C). These results indicated that the uncoating and fusion events of the virions took place prior to the separation of different vRNA segments and the colocalized PB2 and NA vRNAs were the ones co-packaged in the same virus particles. In comparison with mock treated cells, lower numbers of vRNA molecules were imported into the nucleus in ammonium chloride treated cells, indicating that ammonium chloride treatment prohibited nuclear import of most vRNAs (Fig. 3D). Some vRNAs were still able to escape the inhibition and enter the nucleus. Nonetheless, the nuclear PB2 and NA vRNAs detected in the ammonium chloride treated cells exhibited decreasing levels of colocalization over time and the colocalization efficiency was significantly lower than that for the cytoplasmic vRNAs (Fig. 3B&C). These data show that vRNPs become separated once they enter the nucleus and also argue that most of the colocalized PB2 and NA vRNAs detected in the cytoplasm are from the same incoming virions instead of vRNAs from different virions infecting the same cell.

To further understand if the vRNPs released from the virions become separated upon entry to the cytosol or nucleus, infected cells were treated with importazole (IPZ) to block nuclear import of vRNPs. IPZ has been found to interfere with the interactions between importin-β and Ran-GTP, an event critical for the release of imported cargos. Treatment with IPZ, therefore, inhibits the importin-β dependent nuclear import of cargo and retains the imported cargos at the rims of the nuclei [20]. When MDCK cells infected with PR8 virus were treated with IPZ, lower copy numbers of PB2 and NA vRNPs were imported into the nucleus as compared to those in the mock treated cells (Fig. 3D). However, high colocalization efficiency between PB2 and NA was detected in both cytoplasm and nucleus at 20, 40 and 60 minutes post infection. This result suggested that the disassembly of PB2 and NA vRNPs requires nuclear import and occurred after they are released from the nuclear import machinery (Fig. 3A–C). Since there are significantly more separated PB2 and NA vRNPs observed in the mock treated cells than in cells in which the nuclear import of vRNPs is abrogated, we hypothesized that the separated cytoplasmic vRNPs are those that shuttle back into the cytoplasm after nuclear import. We then tested this hypothesis by treating the infected cells with leptomycin B (LMB) which block the export of influenza vRNPs [10]. When the cells were treated with LMB, the copy number of PB2 and NA vRNPs imported into the nucleus was similar to that in the mock infected cells (Fig. 3D) and the colocalization efficiency between the two vRNPs was also comparable in the nuclei of LMB treated and mock treated cells (Fig. 3B). These indicated that the nuclear import of vRNPs in both LMB treated and mock treated cells shared a resembling kinetics. While Crm-1 dependent nuclear export was inhibited by LMB treatment in MDCK cells (Fig. S6), higher proportion of colocalized PB2 and NA vRNAs could be observed in the cytoplasm as compared to those in mock treated cells (Fig. 3A). Quantitative analysis also showed that the colocalization efficiency between PB2 and NA vRNAs was maintained at 70% in cells treated with LMB over time, which was significantly higher than that in the control cells (Fig. 3C). These results suggested that the separated PB2 and NA vRNPs found in the cytoplasm mainly consists of the vRNPs that re-entered the cytoplasm after nuclear import. These further imply that vRNPs are exported individually into the cytoplasm during the early phase of viral infection.

### Colocalization between PB2 and NA in the cytoplasm is microtubule independent

**Figure 4:**
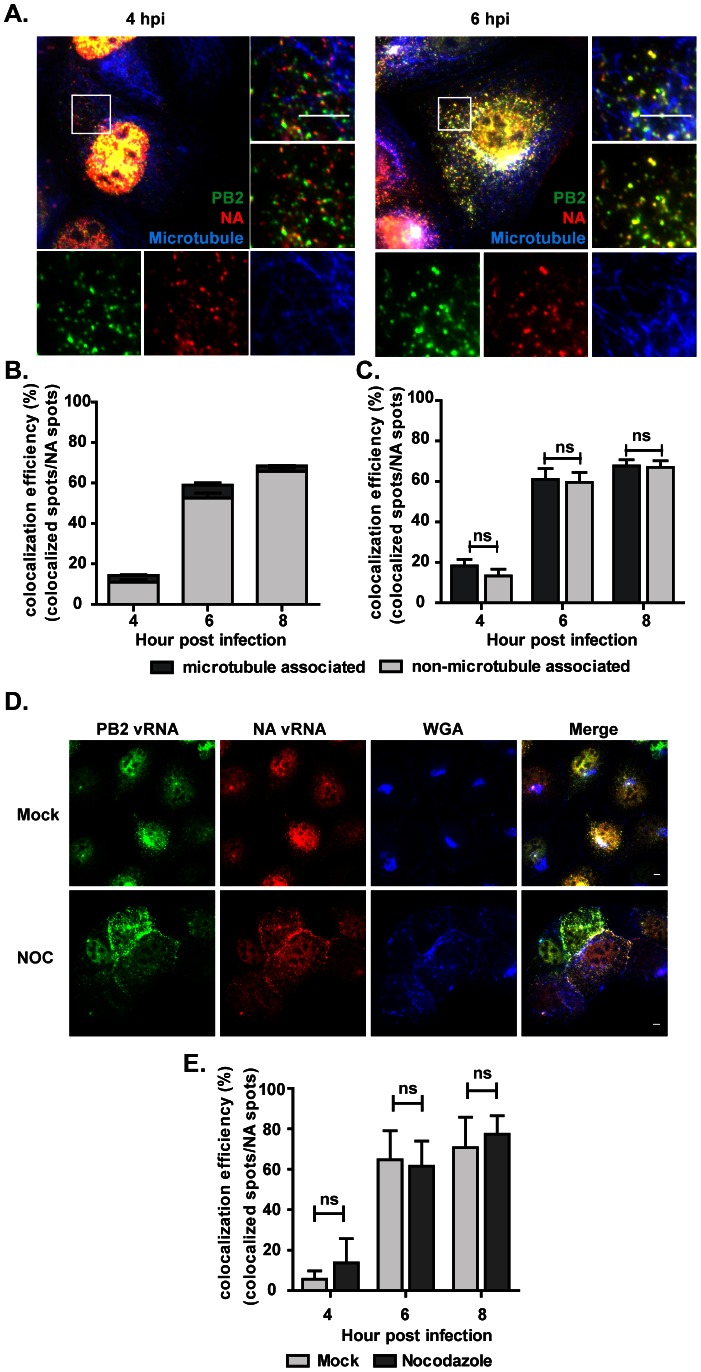
The colocalization of PB2 and NA vRNAs is independent of microtubules. (**A&B**) MDCK cells infected with PR8 virus at MOI = 5 were subjected to immunofluorescence staining of the microtubules (blue) and two-color smFISH using Cy5- or Cy3-fluorophore labeled probes against the PB2 vRNAs (green) or the NA vRNAs (red), respectively. (**A**) Two-dimensional merged images of the microtubules, PB2 and NA vRNAs are shown for cells at 4 and 6 hpi (upper left image in each panel). The enlarged image of the boxed region is shown on the upper right corner; the enlarged images taken in each channel (bottom), and the merged image between the PB2 and NA vRNA spots are also shown (middle, right). Scale bar = 10 µm. (**B**) Quantification of the colocalization between PB2 and NA vRNAs is shown. The proportions of colocalized vRNAs associated with microtubules are represented in dark gray and the colocalized vRNAs that are not colocalized with microtubules are denoted in light gray. The percentages of colocalized vRNAs associated with microtubules are 24.5% at 4 hpi, 10.3% at 6hpi and 4.1% at 8hpi. Error bars represent standard deviation. (**C**) Quantification of the colocalization between PB2 and NA vRNAs that were associated with microtubules (dark gray) or not associated with microtubules (light gray). Error bars represent standard deviations. ns: un-paired t-test no significance. (**D&E**) MDCK cells were infected with PR8 virus at MOI = 5 and treated with nocodazole (NOC) at 1.5 hpi. (**D**) The treated cells were fixed at 8 hpi for smFISH analysis. Images of the Cy5 fluorescence (PB2 vRNAs), Cy3 fluorescence (NA vRNAs), Alexa 488 signal (cell membrane), and a merged image are shown for mock treated or NOC treated cells (Maximum Intensity Projections). Alexa-488 conjugated wheat germ agglutinin (WGA) stains the plasma membrane and Golgi apparatus in the cells. Scale bar = 5 µm. (**E**) Colocalization efficiency of PB2 and NA vRNAs in mock treated and NOC treated cells are shown. For each experimental condition, more than 50 cells were analyzed. Error bars denote standard deviation. ns: un-paired t-test no significance.

Since the results of colocalization analysis of PB2 and NA vRNPs at different time points post infection indicated that vRNPs of different identities colocalized in the cytoplasm, cellular factors that may be involved in this process were further investigated. It has been reported that influenza vRNA trafficking depends on microtubules, and it has been observed that vRNAs accumulated at the microtubule-organization center (MTOC) after their export from the nucleus [17]. Thus, we first analyzed whether microtubules are involved in the colocalization of different vRNP segments by looking at the colocalization of the vRNAs with the microtubule network. To quantify the degree of colocalized vRNAs associated with microtubules, a three-color colocalization analysis was performed. The infected cells were first stained for microtubules using an antibody against tubulin followed by smFISH against the PB2 and NA vRNAs. In Figure 4A, the microtubule is represented in blue while the two vRNAs were represented in green (PB2) and red (NA). If the colocalized vRNAs were associated with microtubules, white signals should be observed. At 4 hpi, the PB2 and NA vRNAs were not colocalized so distinct green and red spots can be observed. It is of note that these vRNAs, albeit not colocalizing, were seen in positions adjacent to the microtubules, suggesting that microtubules may support the transport of individual vRNAs after they were exported from the nucleus. At 6 hpi, the PB2 and NA vRNAs started to colocalize in the cytoplasm. At this time point, white signals were observed at the peri-nuclear region demonstrating the accumulation of PB2 and NA vRNAs at the MTOC. Except for the signals observed in the MTOC regions, the colocalized PB2 and NA vRNAs in the cytoplasm were not located on the microtubule network (Fig. 4A). This suggested that the colocalization of PB2 and NA vRNAs may not happen as they travel along the microtubules. To quantify whether the association between vRNAs is modulated by their location relative to microtubules, we automatically delineated the regions of the three dimensional image stack where the microtubules were located (Fig. S7). The colocalized vRNAs that reside within the contours of these regions were considered to be microtubule associated while the others were not. By counting the number of colocalized spots inside or outside of the contours, the percentage of colocalized vRNAs associated with microtubules can be determined. This analysis showed that small proportions (4–24.5%) of colocalized vRNAs were found to be associated with microtubules, regardless of the increased colocalization efficiency between PB2 and NA vRNAs at 6 and 8 hpi, suggesting that microtubules may not play a major role in the colocalization of different vRNPs (Fig. 4B). We used the same spatial analysis to compare the colocalization efficiency of PB2 and NA vRNAs associated with the microtubules with that of vRNAs that were not associated with the microtubules. No significance difference was observed between the two groups (Fig. 4C). This further indicated that the colocalization of PB2 and NA vRNAs is independent of microtubule association.

To further confirm that an intact microtubule network is not important for PB2 and NA vRNAs to colocalize, we inhibited the polymerization of microtubules with nocodazole in PR8 virus infected cells. MDCK cells infected with PR8 were treated with nocodazole at 1.5 hpi and the cells were then fixed for hybridization at 6 and 8 hpi. Cells were stained with the Alexa488-conjugated wheat germ agglutinin (WGA) to label the plasma membrane and Golgi apparatus. At 6 hpi, large numbers of vRNAs were observed to accumulate at the juxta-nuclear positions in mock treated cells while in nocodazole treated cells, the vRNAs were spread out in the cytoplasm and accumulated toward the lateral plasma membrane (Fig. 4D). This observation was consistent with previous reports showing that microtubules are involved in the apical transport of vRNAs [16]. However, colocalization between the PB2 and NA vRNAs could still be observed in both mock treated and nocodazole treated cells (Fig. 4D). Quantification of the colocalization efficiency between the two vRNAs also showed no difference between the mock treated and nocodazole treated cells (Fig. 4E). This confirms the results from the three-color colocalization analysis that the microtubule network is involved in the destination of trafficking vRNAs but is not involved in the process of vRNA gathering. This also implies that the movement of vRNAs to the MTOC is not essential for PB2 and NA vRNAs to find each other during their journey in the cytoplasm.

### Association of viral RNAs with Rab11 positive organelle plays partial roles in the colocalization of PB2 and NA vRNAs

**Figure 5:**
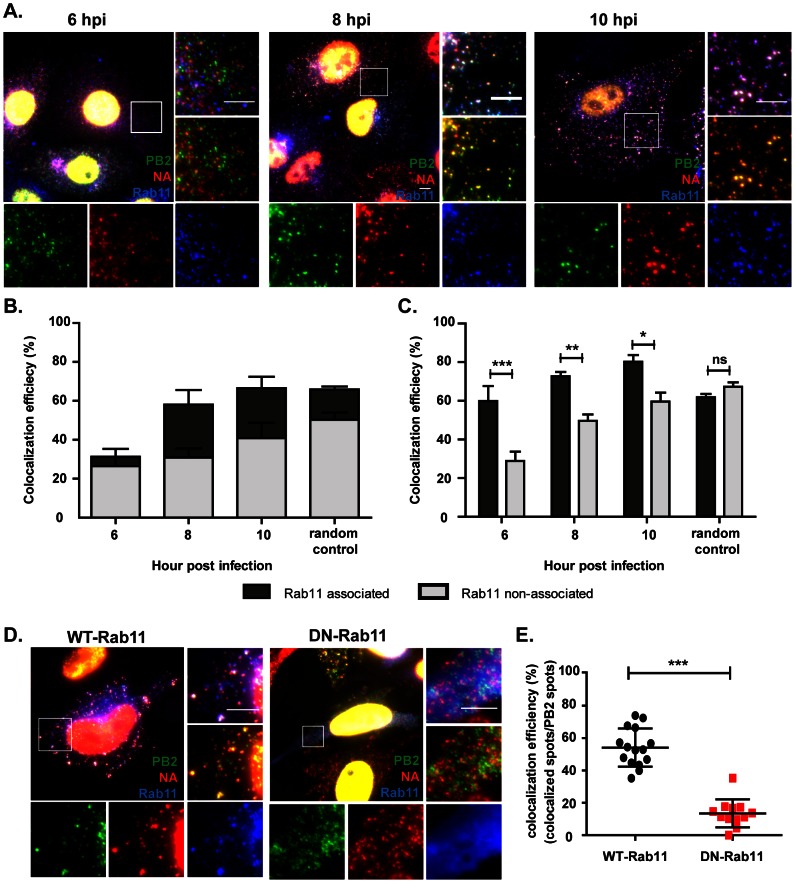
Rab11 is involved in the colocalization of PB2 and NA vRNAs. (**A–C**) A549 cells are infected with PR8 virus at MOI = 5 and were fixed at 6, 8 and 10 hpi. Immunofluorescence staining was performed using anti-Rab11 antibody followed by two-color smFISH using Cy5-labeled PB2 probes and Cy3-labeled NA probes. (**A**) Merged fluorescent images of cells at 6, 8 and 10 hpi are shown on the upper left corners (Maximum Intensity Projection). For each panel, the boxed region is enlarged and shown on the upper right corner. The enlarged images of the PB2 vRNAs (green), NA vRNAs (red), and Rab11 (blue) are shown at the bottom, and the merged image of the PB2 and NA vRNA signals is shown on the middle right. Scale bar = 5 µm. (**B**) Colocalization efficiency of the PB2 and NA vRNA in the cytoplasm at 6, 8 and 10 hpi. The colocalized vRNAs that colocalize with Rab11 are represented in dark gray while the colocalized vRNAs that are not associated with Rab11 are shown in light gray. The percentages of colocalized vRNAs associated with Rab11 are 31.3% at 6hpi, 44.9% at 8hpi, 38.8% at 10hpi and 24% for random control. Error bars represent standard deviation. (**C**) Colocalization efficiency of PB2 and NA vRNAs associated with and not associated with Rab11 in cells at 6, 8 and 10 hpi. The random control represents the colocalization efficiency of PB2 and NA vRNA when the vRNAs were randomly classified into Rab11-associated and Rab11-non-associated groups. Error bars denote standard deviations. ***: t-test p value<0.001, **: t-test p-value<0.01 and *: t-test p value<0.05. (**D&E**) A549 cells were transfected with 1 µg of GFP-rab11-WT or GFP-rab11-DN and infected with PR8 virus at MOI = 5 at 24 hour post transfection. Two-color smFISH using Cy5 probes targeting the PB2 vRNAs and Cy3 probes targeting the NA vRNAs were performed at 8 hpi. (**D**) Images are shown for cells transfected with WT-Rab11 or DN-Rab11 (Maximum Intensity Projections). Magnified images of the boxed areas are shown: the separate images showing PB2 vRNAs (green), NA vRNAs (red) and GFP-tagged rab11 proteins (blue) are at the bottom; a merged image of the PB2 and NA vRNAs is shown at the middle right while the merged image of the three channels is located on the upper right corner of the panel. Scale bar = 5 µm. (**E**) Colocalization of PB2 and NA vRNAs in WT-Rab11 or DN-Rab11 expressing cells. The colocalization efficiency of PB2 and NA vRNAs were analyzed for individual cells that were detected with GFP signal (expressing the GFP-tagged rab11 proteins). The black dots symbolize the colocalization efficiency of vRNAs in WT-Rab11 transfected cells while the red squares symbolize the colocalization efficiency in DN-Rab11 transfected cells. The error bars represent standard deviations. ***: t-test p value<0.001.

It has been reported that influenza vRNAs travel to the plasma membrane in a Rab11 dependent manner [15], [16], [17], [19]. The colocalization of vRNAs in the cytoplasm at late time points post infection may occur when the vRNPs are loaded onto Rab11 positive vesicles. We therefore tested whether the transport of vRNAs with Rab11 positive organelles is critical for the colocalization of PB2 and NA vRNAs. In Figure 5A, PR8 virus infected A549 cells were hybridized with probes against the PB2 and NA vRNAs and were immunostained against Rab11 proteins. Since the antibody against Rab11 specifically detects human Rab11 molecules, two-color smFISH and immunostaining against Rab11 were performed in virus infected A549 cells. It was observed that colocalization between PB2 and NA vRNAs exhibited slower kinetics in A549 cells compared to that detected in MDCKs (Fig. S8), therefore the spatial-temporal relationships between colocalized vRNAs and Rab11 were then determined at 6, 8 and 10 hour post infection (instead of 4, 6 and 8 hour post infection in MDCK cells). At 6 hpi, the PB2 and NA vRNAs were not colocalized nor did they spatially coincide with Rab11 particles. However, at 8 and 10 hpi, PB2 and NA vRNAs displayed a high colocalization efficiency (appearance of yellow puncta in the two color FISH images) and these colocalized vRNAs were found associated with Rab11 particles, giving rise to distinct white puncta when FISH and immunostaining images were merged together. This suggests that the colocalization of PB2 and NA vRNAs likely happens when the two vRNAs are loaded onto the same Rab11-positive vesicle. To further quantify the proportion of colocalized vRNAs that associated with Rab11, a three-color colocalization analysis similar to the one described for microtubules was performed. The results show that the portion of Rab11-associated vRNPs which colocalize increases from 13.2% at 6 hpi to 44.9% at 8hpi, corresponding to the increase in the colocalization efficiency between PB2 and NA vRNAs from 31% at 6 hpi to 58% at 8 hpi (Fig. 5B). Furthermore, we found that the colocalization efficiency of the Rab11-associated PB2 and NA vRNAs was significantly higher than that of non Rab11 associated vRNAs (Fig. 5C). These results suggest that the localization of vRNAs with Rab11 enhances the degree of colocalization between PB2 and NA vRNAs.

To further demonstrate the involvement of Rab11 in the colocalization of PB2 and NA vRNAs, A549 cells were transfected with GFP tagged Rab11 in either the wild type or dominant negative forms followed by PR8 virus infection. The degree of colocalization between PB2 and NA vRNAs was compared in cells that expressed the wild type Rab11 (WT-Rab11) and cells that expressed the dominant negative Rab11 (DN-Rab11). Figure 5D shows that PB2 vRNAs colocalized with NA vRNAs in WT-Rab11 transfected cells and the colocalized vRNAs were located to Rab11 as well. On the other hand, the cells transfected with DN-Rab11 show a disperse distribution of PB2 and NA vRNAs in the cytoplasm; no colocalization was seen between the vRNAs. The DN-Rab11 proteins were also diffused in the cytoplasm, similar to what was previously reported [15]. We observed a large accumulation of vRNAs in and around the nucleus in DN-Rab11 protein expressing cells; we adjusted the contrast of the displayed images to correctly represent the vRNA particles in the cytoplasm. When a quantitative analysis was performed, the colocalization efficiency of the PB2 and NA vRNAs in the cytoplasm was significantly higher in WT-Rab11 transfected cells than in the cells expressing DN-Rab11 (Fig. 5E). These results further demonstrate the importance of Rab11 in facilitating the colocalization of different vRNAs during influenza virus infection. Together the data show that vRNAs of different segments colocalize before they reach the plasma membrane and the Rab11 positive organelles may serve as a platform for the gathering of different vRNAs as they travel to the site of assembly.

### Colocalization between PB2 and NA vRNAs in the cytoplasm is HA and M2 independent

**Figure 6:**
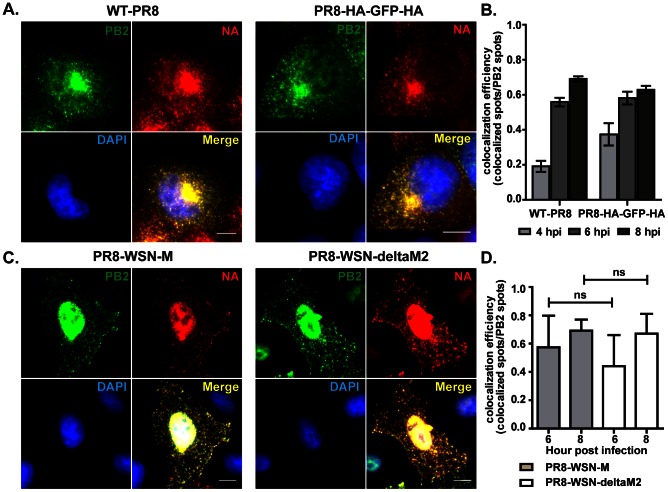
The colocalization of PB2 and NA vRNAs is independent of expression of viral proteins HA and M2. (**A&B**) MDCK cells were infected with either wild type PR8 (WT-PR8) or PR8-HA-GFP-HA viruses and fixed for two-color smFISH analysis against PB2 and NA vRNAs at 4, 6 and 8 hpi. (**A**) DAPI signal (blue), Cy5 fluorescence for PB2 vRNAs (green) and Cy3 fluorescence for NA vRNAs (red) for the cells infected with WT-PR8 and PR8-HA-GPF-HA viruses at 8 hpi (Maximum Intensity Projection). Scale bar = 10 µm. (**B**) Colocalization efficiency between PB2 and NA vRNAs in the cytoplasm of cells infected with either WT-RP8 or PR8-HA-GFP-HA viruses is shown. Error bars denote standard deviation. ns: t-test p value>0.05; no significance. (**C&D**) MDCK cells were infected with either PR8-WSN-M or PR8-WSN-ΔM2 at MOI = 5 and two-color FISH targeting the PB2 (Cy5 labeling) and NA (Cy3 labeling) vRNAs was performed at 6 and 8 hpi. (**C**) Fluorescent images of infected cells hybridized with Cy5 PB2 probes (green) and Cy3 NA probes (red) at 8 hpi (Maximum Intensity Projections). DAPI staining (blue) stains the nucleus of the cell. Scale bar = 10 µm. (**D**) Colocalization efficiency of PB2 and NA vRNAs in the cytoplasm of cells infected with PR8-WSN-M or PR8-WSN-ΔM2. Error bars denote standard deviations. ns: un-paired t-test no significance.

The viral proteins hemagglutinin and M2, specifically the cytoplasmic tail of M2, have been reported to be involved in the assembly of budding virions [21], [22]. We therefore tested whether HA and M2 are involved in the colocalization of vRNAs during their travel to the plasma membrane. A recombinant PR8 virus that lacks the HA ORF, PR8-HA-GFP-HA virus, was generated in MDCK cells which constitutively express the HA protein (MDCK-HA). The HA ORF of this virus is substituted with the ORF of the GFP gene. To test the role of HA protein in the colocalization of vRNAs, MDCK cells were infected with either the wild type PR8 (WT-PR8) or PR8-HA-GFP-HA viruses followed by smFISH and colocalization analysis of the PB2 and NA vRNAs at 4, 6 and 8 hpi. Since the PR8-HA-GFP-HA virus lacked the HA ORF, no HA protein could be encoded and the involvement of HA protein in this process can then be assessed. The PR8-HA-GFP-HA virus showed similar kinetics in vRNA replication and vRNA export as compared to the wild type PR8 virus in MDCK cells (data not shown). Comparable kinetics for the colocalization between PB2 and NA vRNAs were also observed for WT-PR8 and the PR8-HA-GFP-HA viruses (Fig. 6A&B). These results demonstrated that the expression of HA protein was not critical for the colocalization of different vRNAs in an infected cell.

The role of M2 protein was assessed with a similar strategy. Two recombinant viruses in the PR8 virus background were generated [23]. The PR8-WSN-M contains the full-length M segment from the WSN virus, so both M1 and M2 can be produced during infection. The PR8-WSN-ΔM2 virus lacks the M2 ORF, so it is grown in MDCK cells that expressed the M2 protein. The colocalization efficiency between the PB2 and NA vRNAs was compared between MDCK cells infected with PR8-WSN-M and PR8-WSN-ΔM2 viruses. No significant difference was observed between the PR8-WSN-M and PR8-WSN-ΔM2 virus infected cells. These results suggested that the expression of M2 protein did not play an essential role in the colocalization between PB2 and NA vRNAs (Fig. 6C&D).

## Discussion

**Figure 7:**
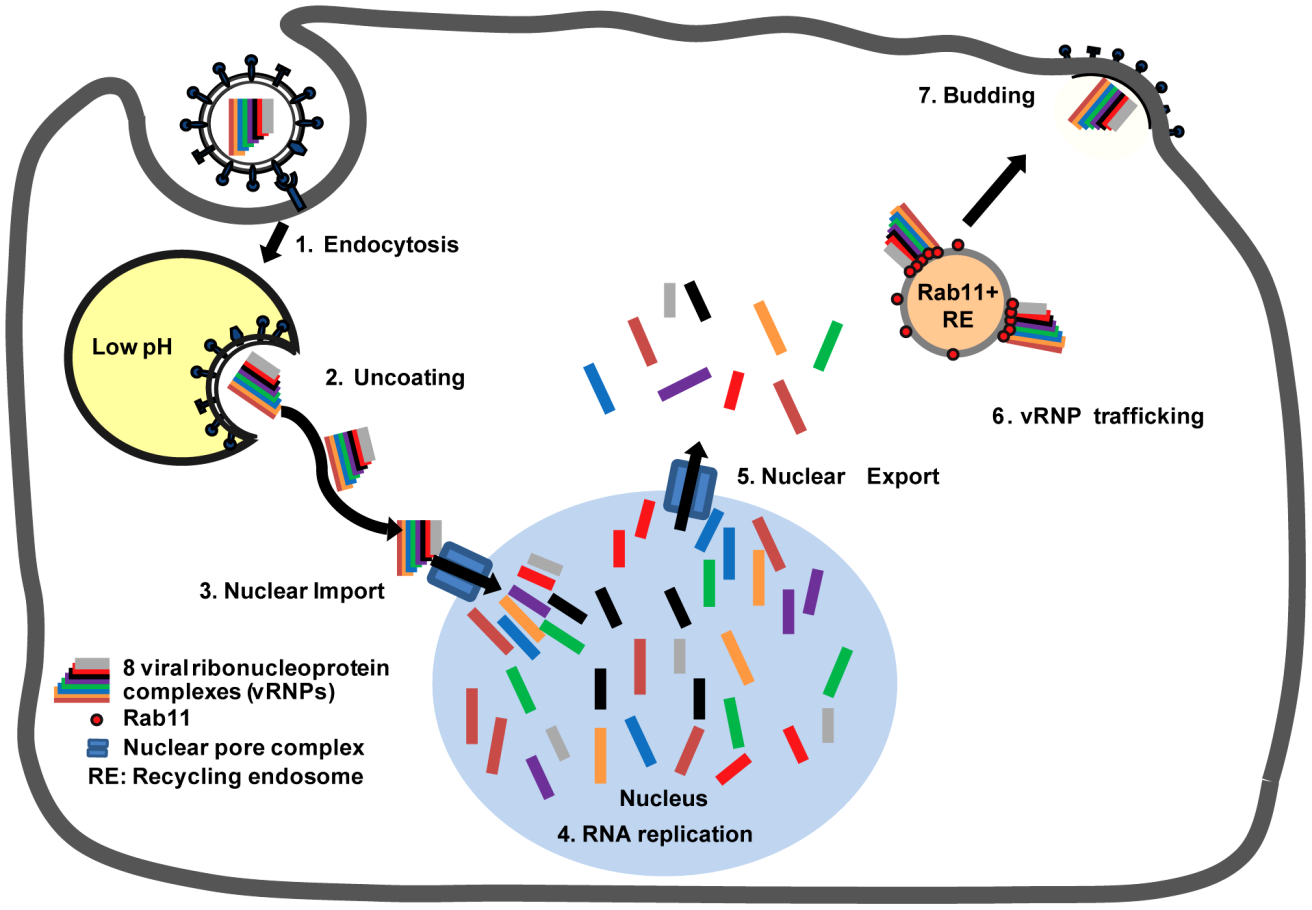
Proposed model for the disassembly and colocalization of different vRNA segments in an infected cell. (**1**) An influenza virus particle enters the cell through the endocytic pathway. (**2**) Acidification of the endosome allows fusion of viral envelope with the endosomal membrane and the release of vRNPs into the cytosol. The vRNPs packaged in the virus particles remain tightly associated during their transport towards the nucleus. (**3**) Upon nuclear import, the associated vRNPs of different segments disassemble in the nucleus (Results in Figure 3) and (**4**) different vRNAs replicate in different areas of the nucleus and no segment-specific accumulation of vRNAs is observed (Results in Figure 2&3). (**5**) The newly synthesized vRNPs are exported into the cytoplasm individually and different segments are not associated with each other shortly after their nuclear export (Results in Figure 2&3). (**6**) During the vRNP trafficking to the plasma membrane, the vRNPs are loaded onto recycling endosomes through the association with Rab11 [15], [16], [17]. The vRNPs of different segments then start to colocalize with each other and travel together towards the apical plasma membrane as a pre-formed complex of eight different vRNPs. (**7**) As the complex reaches the plasma membrane, the eight different vRNPs are then packaged as a whole into the budding virions.

In this study, we have analyzed the disassembly and the subsequent assembly of influenza vRNPs segments in virus-infected cells using smFISH. We show that vRNPs of different segments remain associated after their release from the incoming virions and they travel as a package to the nuclear membrane. Newly synthesized vRNPs of different segments do not occupy the same space in the nucleus and they are likely exported individually into the cytoplasm because colocalization of the exported vRNPs of different segments is not observed during the early stages of infection. Different viral RNPs colocalize in the cytoplasm at later stages during infection (6–8 hpi in MDCK cells) and they are often found to be associated with Rab11-recycling endosomes. These results provide evidence that vRNPs belonging to different segments follow the same trafficking route and the selection for the correct combination of the eight vRNA segments likely takes place before the vRNPs reach the cell surface (Fig. 7).

The detection of vRNPs as early as 20 minutes post infection demonstrates the high sensitivity of the smFISH methodology, which allows analysis of the spatial relationship of the vRNPs during the viral entry process. When uncoating of the incoming virus particles was blocked by increasing the pH in the endosomal compartments, the PB2 and NA vRNAs were found to colocalize with very high efficiency. These results indicate that the colocalized vRNPs originate from the same virions, in accordance to the previous report that influenza virus particles package their eight different vRNAs with high efficiency [4]. It was suggested earlier that vRNPs of different segments travel as a package before nuclear import, because when multi-nucleated skeletal myofibers were infected with influenza viruses at low MOI, viral RNAs of different segments were observed to enter the same nucleus [24]. With smFISH analysis, quantification of the level of colocalization of different vRNPs became possible. When infected cells were treated with importazole, a recently discovered compound interfering with importin-β and Ran-GTP interactions [20], the vRNPs of different segments stayed colocalized. This implied that after the vRNPs were released from the virions, they remained associated as they travel to the nucleus. In addition, it was observed that imported vRNPs of different segments remained colocalized upon IPZ treatment. Since IPZ prohibit the release of imported cargo from the cellular nuclear import machinery, it is possible that the release of vRNP complex from the import machinery is necessary for the separation of different segments or the initiation of vRNA replication. Three-dimensional reconstruction of viral RNPs inside budding virions revealed an electron dense platform at the leading tip of the vRNPs, suggesting that there are interactions among vRNPs in this region [3], [5]. Recent studies using RNA mobility shift assays of *in-vitro* transcribed vRNAs suggested that specific RNA/RNA interactions among vRNAs exist and that they may participate in the process of genome selection [5], [25]. However, further investigation is required to understand the detailed mechanism by which the eight vRNPs are held together during their travel in the cell.

The replication of vRNAs of cytoplasmic negative-sense RNA viruses has been found to occur in virally induced compartments, such as Negri bodies for rabies virus [26], or inclusion bodies for vesicular somatitis virus [27] and Ebola virus [28]. However, no specialized compartments have been observed for the synthesis of influenza RNAs in the nucleus. Indeed, we observed that the newly synthesized vRNAs were distributed throughout the nucleoplasm, except the nucleoli, similar to what was shown for the distribution of nucleoproteins in the nucleus [9]. The observation of separated vRNAs of different segments may indicate that the nuclear import of vRNPs drove the disassembly of the vRNP complex and each segment was distributed to a different position for transcription and replication. It is also possible that newly synthesized vRNA quickly diffused from the site of replication where different vRNA segments are in close proximity. A polymerase-mutant virus would be helpful to further elucidate the behavior of vRNAs in the nucleus. Moreover, separated newly synthesized vRNPs were detectable in the cytoplasm at the time when accumulation of vRNPs and the increased efficiency of colocalization between two different vRNPs in the nucleus were observed. This arguably suggests that the progeny vRNPs are exported individually from the nucleus and that the RNP complexes of colocalized segments only form in the cytoplasm.

The Rab11 recycling endosome system has been shown to be involved in the transport of vRNPs from the peri-nuclear regions to the apical plasma membrane [15], [16], [17]. It has also been shown that vRNPs were actively transported on microtubules [14] and an intact microtubule network was required for the targeting of Rab11 recycling endosomes to the apical surface [16], [17]. In our study, we show that microtubules are dispensable for the colocalization of different vRNPs while they are necessary for the correct membrane targeting of vRNPs. The three-color smFISH analysis shows that the interaction of vRNPs with Rab11 increases the colocalization efficiency of different vRNA segments, demonstrating that Rab11 plays a role in facilitating the colocalization between different vRNA segments. Thus, it is suggested that the interaction of vRNPs with Rab11 concentrates the vRNPs of different segments onto vesicle membranes and enhances the possibility of different vRNPs coming together. In fact, a high degree of colocalization between PB2 and NA vRNA is usually detected in cells in which accumulations of vRNPs at the peri-nuclear regions are observed, a proposed location where vRNPs are loaded onto Rab11-positive vesicles [16], [17]. We therefore propose that Rab11 recycling endosomes serve as membrane platforms which help vRNPs of different segments to find their partners. It should be noted, however, that colocalized vRNPs which were not associating with Rab11 could be observed (Fig. 6B). This indicates that there may be other factors, such as the packaging signals of vRNAs, determining the colocalization between different vRNPs (independent of Rab11 interactions). It is also possible that these colocalized vRNPs were disassociated from Rab11 as they were ready to be packaged into the virus particles. Taken together, it is likely that the selection of different vRNA segments to be co-packaged into virions takes place during vRNP trafficking to the plasma membrane.

The efficient incorporation of influenza viral genome into virions has been linked to the cytoplasmic tail of the virus transmembrane proteins: HA, NA and M2 [21], [29], [30]. Mutant influenza A virus lacking the HA and NA cytoplasmic tail showed reduced viral RNA to viral protein content; it was also suggested that this mutant virus contain more non-infectious virus particles (without full complemented genome) than the wild type viruses [29]. In addition, it has been demonstrated that truncations or mutations in the cytoplasmic tail of the M2 protein lowered the level of virion associated NP proteins and vRNAs [21], [30], [31], [32]. This effect has been linked to the interactions between the M1 protein and the M2 cytoplasmic tail, suggesting that M2 is responsible for the recruitment of M1-vRNP complexes to the virion budding sites [31], [33]. With these lines of evidence, one would postulate that the assembly of vRNPs happens at the plasma membrane. However, our study using recombinant viruses lacking the entire HA or M2 protein indicates that the colocalization between different vRNP segments is independent of HA and M2 recognition (Fig. 6), further confirm other data in this present study demonstrating that the different vRNP segments assembled prior to their incorporation into budding virions. Since it has been shown that the majority of influenza viruses package the complete set of eight vRNA segments [4] and physically associated, well-ordered eight vRNPs are observed in budding virions [3], [5], [25], it is proposed that an interlinked complex of eight vRNPs formed before them being packaged [3], [34].Even though the formation of the super-complex cannot be detected with the current smFISH experiments, the colocalization between different vRNA segments detected in the cytoplasm suggested that the process of complex formation might occur during vRNA trafficking, providing an additional hint to the current hypothesis that the selected eight vRNPs might be packaged as a complex into budding virion.

In conclusion, we have shown with smFISH and colocalization analyses that different vRNA segments in influenza virus-infected cells colocalized in the cytoplasm before they reached the plasma membrane. These findings shed light on the selection process of influenza vRNPs packaging [3], [34], however, further investigations are required to fully identify the biological principles that govern the associations between the different vRNPs.

## Materials and Methods

### Cells, viruses, plasmids and antibodies

Madin-Darby canine kidney (MDCK) epithelial cells were maintained in Modified Eagle's Medium (MEM) (Gibco, Invitrogen) supplemented with 10% fetal bovine serum (FBS). Adenocarcinomic human alveolar basal epithelial cells (A549) were grown in Dulbecco's Modified Eagle's Medium (DMEM) (Gibco, Invitrogen) supplemented with 10% FBS. Both cell lines were incubated at 37°C with 5% CO_2_. Influenza A/Puerto Rico/8/34 (PR8) and PR8 cH9/1 virus strains were grown in 10-day old embryonic chicken eggs as previously described [35]. Recombinant PR8-HA-GFP-HA virus was grown in HA-MDCK cells [36]; PR8-WSN-M and PR8-WSN-ΔM2 viruses were grown in M2-complementing MDCK cell lines [23] (kindly provided by Dr. Randy Albrecht). Plasmids GFP-rab11 WT and GFP-rab11 DN were purchased from Addgene (Addgene plasmid 12674 and 12678) [37]. The following antibodies were used: a rat monoclonal anti-α-tubulin antibody (Abcam), a rabbit anti-Rab11 polyclonal antibody (Invitrogen), Alexa Fluor 488-conjugated donkey anti-rabbit IgG (H+L) antibody and an Alexa Fluor 488-conjugated goat anti-rat IgG (H+L) antibody (Molecular probes).

### Generation of PR8-HA-GFP-HA virus

The reverse genetics method for generating recombinant PR8-HA-GFP-HA virus was as described previously [36]. The pDZ plasmid expressing GFP ORF flanked by the HA packaging signals was constructed as previously reported [38].

### Virus infection

Infection of MDCK cells or A549 cells with influenza viruses was performed as previously described [39]. To synchronize virus entry, the cells were first incubated on ice for 5 min and then incubated with viruses diluted in infection media (phosphate buffered saline (PBS) supplemented with 1% bovine albumin (BSA) and 1% penicillin-streptomycin) on ice for 60 min. After virus adsorption, the cells were washed and the post-infection media (DMEM supplemented with 0.3% BSA, 1% penicillin-streptomycin, and 1 µg/ml TPCK (L-1-tosylamide-2-phenylethyl chloromethyl ketone)-trypsin) pre-warmed to 37°C was added immediately to the cells. The cells were then transferred to 37°C to allow virus entry. For cells treated with drugs during infection, the compounds were present in both the infection media and post-infection media at the following concentrations: 20 mM for ammonium chloride; 40 ng/ml for leptomycin B (Sigma); 31.8 µg/ml for importazole (Sigma) and 20 µg/ml for nocodazole (Sigma).

### Transfection

5×10^4^ A549 cells were transfected with 1 µg of GFP-rab11-WT or GFP-rab11-DN plasmid using lipofectamine 2000 reagent according to manufacture protocol (Invitrogen). Cover slips were coated with fibronectin (Sigma) by incubating the cover slips in 50 µg/ml of fibronectin in PBS for 45 min at RT and rinse them once with PBS. The transfected cells were plated onto the fibronectin coated coverslips and incubated at 37°C for 24 hours before virus infection.

### Single-molecule sensitivity RNA fluorescence *in situ* hybridization

RNA fluorescence *in situ* hybridization (FISH) was performed according to published protocols with some modifications [40], [41], [42]. The probes used were single-stranded DNA oligos (20 nucleotides) each labeled with one fluorophore (Cy3 or Cy5) (BioSearch, Text S1). Cells were plated onto poly-lysine coated coverslips (BD Biosciences) at a density of 5×10^4^ cells/well and grown overnight at 37°C. At certain time points post infection, the cells were washed once with ice-cold PBSM (1×PBS, 5 mM MgCl_2_), followed by fixation with 4% paraformaldehyde in PBSM for 10 min at room temperature (RT). After a brief wash with ice-cold PBSM, the cells were permeabilized with 0.5% Triton X-100 in PBSM for 1 min at RT. The cells were then washed with PBSM and incubated in 2XSSC (300 mM sodium chloride, 30 mM sodium citrate) with 10% formamide for 5 min before hybridization. To detect viral RNAs, 4 µM of labeled probes in 40 µl of hybridization buffer (10% dextran sulfate, 2 mM vanadyl ribonucleoside complexes (VRC, New England BioLabs), 0.02% RNAse-free BSA, 50 µg E. coli tRNA, 2XSSC, 10% formamide) was used for each sample. Hybridization was carried out in humidified chambers maintained at 37°C for 16 hours. The samples were then washed twice with 10% formamide 2×SSC supplemented with 2 mM VRC for 30 min at 37°C. Nuclear staining using 0.5 µg/ml of DAPI was performed afterwards and the coverslips were mounted in ProLong Gold antifade mounting media (Invitrogen). The cured samples were subjected to microscopy examination or were stored at −20°C. For samples used for both immunofluorescence and FISH analyses, cells were first blocked with 1% BSA in PBSM for 1 hour at RT after fixation and permeabilization. The coverslips were then subjected to primary and secondary antibody staining in 1% BSA in PBS followed by another fixation step with 4% paraformaldehyde for 10 min. The cells were then washed once with PBSM and equilibrated with 10% formamide 2XSSC for 10 min before the *in situ* hybridization procedures.

### Image acquisition

Cells were placed on a Zeiss Axioplan2IE microscope equipped with a 100×, 1.4 numerical aperture (NA) oil-immersion objective (Zeiss) and a Zeiss AxioCam MRm camera. Cells were imaged using 200 nm z-dimension axis steps across a range of approximately 4 µm.

### Image analysis

We performed subpixel localization and intensity quantification of the spots using custom-designed MatLab (Mathworks) programs which were previously described [43]. We used a custom written Matlab program for colocalization quantification. After the center positions of fluorescence spots in each channel were identified in 3-dimensional space using the spot detection program, the distances between spots of one color with their nearest neighboring spots of the other color were calculated. Colocalized events were assigned when the distance between the closest spots of different colors were within 2.5 pixels (255 nm). The value of this distance threshold was determined by comparing the distances between the nearest spots of different colors detecting the same vRNA molecule and those detecting RNA molecules that do not colocalized. The 2.5 pixels (255 nm) range was used to maximize detection of colocalization events while minimizing false positives (see Fig. S2 for further explanations). Within each image, colocalization between spots was separately quantified in various regions of interest (such as nucleus and cytoplasm) using 2D and 3D mask-images. Mask Images were generated in various ways. Individual cells were manually delineated using a plugin written for the Image J program (NIH). Nuclei were segmented using an automatic intensity threshold of a maximum intensity projection of the DAPI image stack. The microtubules and Rab11 immunofluorescence image stacks were subjected to a custom-written automated segmentation tool to generate 3D mask image stacks for colocalization analysis. Briefly, the 3D stack was first smoothed using a bandpass filter before applying an automated threshold using Otsu's Method. The result was a binary 3D image stack in which white voxels corresponded to microtubules (resp. Rab11 particles) while the rest of the 3D space was left black. Microtubules image stacks were deconvolved prior to applying the segmentation algorithm using AutoQuant X2 AutoDeblur software (Media Cybernetics). Custom written software is available upon request.

## Supporting Information

Figure S1 **Specificity of single-molecule sensitivity FISH analysis of influenza viral RNAs.** (**A**) MDCK cells were infected with PR8 virus at MOI = 5. DAPI signal and Cy5 fluorescence from sm-FISH probes targeting the PB2 vRNAs in mock infected and PR8 virus infected cells at 4 hpi are shown in 2D images constructed using maximum-intensity projections. Scale bar = 10 µm. (**B**) MDCK cells were infected with PR8 virus at MOI = 5 and hybridized with 48 Cy5 labeled probes targeting the PB2 vRNAs. Histogram of the fluorescence spots intensity at 1 hour post infected is shown. The spot intensity distribution displays a well-defined single peak characteristic of single molecules. Black circles: data; red line: Gaussian distribution fits of the data. (**C**) MDCK cells were infected with either PR8 or PR8 cH9/1 virus (containing the HA ORF expressing the head region of the H9 subtype) for 6 hours before smFISH was performed using Cy3 labeled probes against the HA1 vRNAs and Cy5 labeled probes against the HA9 vRNAs. Maximum intensity merges of a pair of z-stack images taken in the Cy3 channel (left, red) and the Cy5 channel (right, green) are shown. DAPI staining (blue) stains the nuclei in the cells. Scale bar = 25 µm.
(TIF)

Figure S2 **Empirical determination of the maximum distance threshold between the centers of spots which defines colocalization.** (**A**) The distances between the centers of colocalized spots were empirically determined by using two probe sets targeting different regions of the same viral RNA. Two different regions of NA vRNA were bound by two probe sets, one labeled with Cy3 fluorophore and the other labeled with Cy5 fluorophore. Z-stack images were taken in both fluorescence channels and the centers for the Cy3 spots and Cy5 spots were localized in 3D space using the spot detection program. The distances between the Cy3 spots and their nearest-neighboring Cy5 spots were measured. The Distributions of the distances between the neighboring spots of different colors is shown here. The distances between the centers of the Cy3 and Cy5 spots were found mainly within 1 pixel (102 nm) and spread to approximately 2.5 pixels (255 nm). Since electron microscopy analysis suggested the packaged vRNPs range from 80 nm to 100 nm (the distances between Cy3 and Cy5 spots are theoretically within 1 pixel), the nearest neighbor distances measured here likely represent the accuracy of measurement of the same diffraction-limited object in Cy3 and Cy5 channels. We therefore define spots as being colocalized if their centers are within 2.5 pixels. (**B**) In contrast to colocalizing spots, the distribution of nearest neighbor distances between the NA vRNAs (Cy5 spots) and β-actin mRNA (Cy3 spots) in infected cells shows that the nearest-neighbor distances range from 1 pixel to 10 pixels (1020 nm), with most of the spots clustered within a range from 3.5 pixels to 6 pixels. The red dashed lines show the 2.5 pixels distance threshold to define colocalization of spots in the quantitative colocalization analysis.
(TIF)

Figure S3 **Distribution of vRNPs in the cytoplasm.** Maximum intensity merge images of MDCK cells infected with PR8 virus at 2, 3 (**A**) and 6 (**B**) hpi. The cells were probed against PB2 vRNAs (green), NA vRNAs (red) and stained with DAPI (blue) to define the nuclear regions. The white arrows show the exported vRNAs detected in the cytoplasm. The red arrows indicate the peri-nuclear accumulations of vRNAs. Scale bar = 10 µm.
(TIF)

Figure S4 **Growth kinetics of PR8 virus in MDCK cells.** MDCK cells were infected with PR8 virus at MOI = 5. Supernatant of the infected MDCK cells were collected at 0, 1, 2, 4, 7, 10 and 12 hours post infection. The virus particles released into the supernatant were titered using standard plaque assays. The titer of the released virus particles at each time point is shown.
(TIFF)

Figure S5 **The colocalization of vRNPs of different identities can be observed at 6 hpi.** MDCK cells were infected with PR8 virus at MOI = 5 and sm-FISH and colocalization analyses were performed for different pairs of vRNPs. Quantification of the colocalization efficiency between the indicated vRNA pairs is shown. Low localization efficiency is observed at 4 hpi for all the vRNA pairs tested while elevated colocalization efficiency of these vRNA pairs is detected at 6 hpi, demonstrating similar kinetics.
(TIF)

Figure S6 **Positive control of LMB treatment on MDCK cells.** As a positive control for the effect of LMB, we examined the nuclear-cytoplasmic shuttling protein MAP kinase (MEK) [44] in MDCK cells. The cells were first serum starved for 16 hours and incubated in infection media with 40 ng/ml of LMB for 1 hour (the same concentrations as virus infection was performed). The cells were then fixed with 4% formaldehyde for 10 min and immunofluorescence against the MEK protein using anti-MEK antibody (1∶100, Abcam). The fluorescence images of the MEK protein, the nuclei and the merged are shown. Nuclear accumulation of MEK in LMB treated cells reflects impaired export, whereas non treated cells display a clear cytoplasm localization pattern for MEK. Scale bar = 25 µm.
(TIF)

Figure S7 **Automatic Segmentation of Microtubules and Rab11 Particles.** **Top:** Microtubule Segmentation Example. Left, representative plane from a deconvolved image stack of microtubules labeled with an antibody against tubulin. Center, mask image showing the output of the automated segmentation algorithm for the plane on the left. Right, Overlay of data (green) and segmentation result (red). **Bottom:** Rab11 Particles Segmentation Example. Left, Maximum Intensity Projection of an image stack of Rab11 particles labeled with antibody against Rab11. Center, Maximum Intensity Projection of the output of the automated segmentation algorithm for the image stack represented on the left. Right, overlay of data (green) and segmentation results (red). Scale Bar: 10 µm.
(TIF)

Figure S8 **The colocalization of PB2 and NA vRNAs in A549 cells.** A549 cells were infected with PR8 virus at MOI = 5 and sm-FISH and colocalization analyses were performed for PB2 and NA vRNAs (Cy5 labeled PB2 and Cy3 labeled NA vRNAs). Quantification of the colocalization efficiency between the two vRNAs is shown. Low localization efficiency is observed at 4 hpi and 6 hpi while elevated colocalization efficiency is detected at 8 hpi and 10 hpi, demonstrating a delayed kinetics compared to that in MDCK cells (Fig. 2C and Fig. 4B).
(TIF)

Text S1 **Probes sequences used to target vRNA or cellular mRNA in influenza virus infected cells.** Sequences for each probe used to target influenza vRNA or cellular mRNA are listed here.
(DOCX)
