# Correction: Colocalization of Different Influenza Viral RNA Segments in the Cytoplasm before Viral Budding as Shown by Single-molecule Sensitivity FISH Analysis

**DOI:** 10.1371/annotation/8f53e7f2-2348-436f-b37e-a883a01e9bbd

**Published:** 2013-07-22

**Authors:** Yi-ying Chou, Nicholas S. Heaton, Qinshan Gao, Peter Palese, Robert H. Singer, Timothée Lionnet

The fifth author's name is missing the middle initial. The correct name is: Robert H. Singer. The correct citation is: Chou Y-y, Heaton NS, Gao Q, Palese P, Singer RH, et al. (2013) Colocalization of Different Influenza Viral RNA Segments in the Cytoplasm before Viral Budding as Shown by Single-molecule Sensitivity FISH Analysis. PLoS Pathog 9(5): e1003358. doi:10.1371/journal.ppat.1003358

