# Supplementary figures and images for: Colocalization of Different Influenza Viral RNA Segments in the Cytoplasm before Viral Budding as Shown by Single-molecule Sensitivity FISH Analysis
Source: PLoS Pathog. 2013 May 9;9(5):e1003358. doi: 10.1371/journal.ppat.1003358 (PMC3649991; doi:10.1371/journal.ppat.1003358)

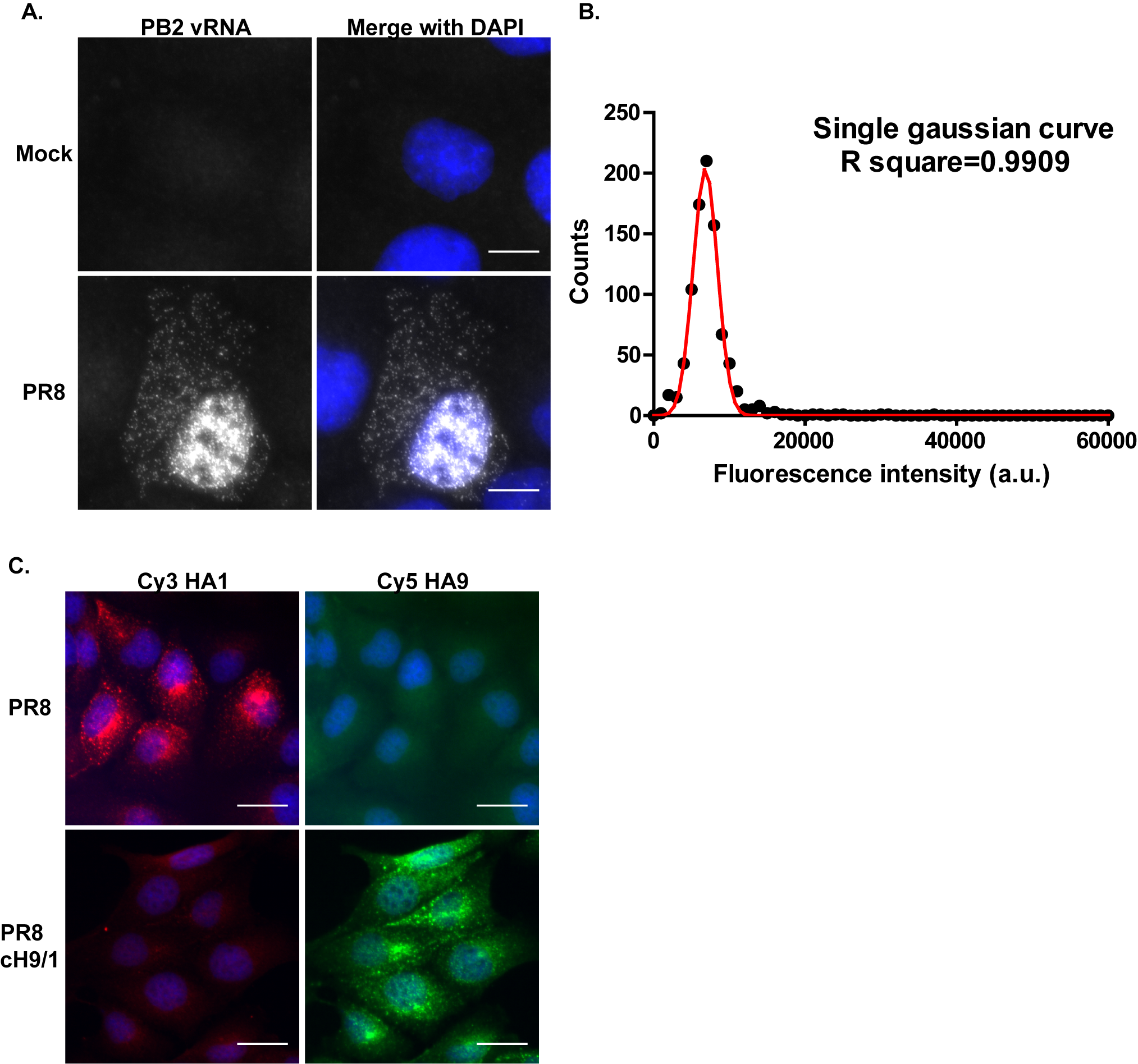

Supplement: Figure S1 — Specificity of single-molecule sensitivity FISH analysis of influenza viral RNAs. (A) MDCK cells were infected with PR8 virus at MOI = 5. DAPI signal and Cy5 fluorescence from sm-FISH probes targeting the PB2 vRNAs in mock infected and PR8 virus infected cells at 4 hpi are shown in 2D images constructed using maximum-intensity projections. Scale bar = 10 µm. (B) MDCK cells were infected with PR8 virus at MOI = 5 and hybridized with 48 Cy5 labeled probes targeting the PB2 vRNAs. Histogram of the fluorescence spots intensity at 1 hour post infected is shown. The spot intensity distribution displays a well-defined single peak characteristic of single molecules. Black circles: data; red line: Gaussian distribution fits of the data. (C) MDCK cells were infected with either PR8 or PR8 cH9/1 virus (containing the HA ORF expressing the head region of the H9 subtype) for 6 hours before smFISH was performed using Cy3 labeled probes against the HA1 vRNAs and Cy5 labeled probes against the HA9 vRNAs. Maximum intensity merges of a pair of z-stack images taken in the Cy3 channel (left, red) and the Cy5 channel (right, green) are shown. DAPI staining (blue) stains the nuclei in the cells. Scale bar = 25 µm. (TIF) [file ppat.1003358.s001.tif]

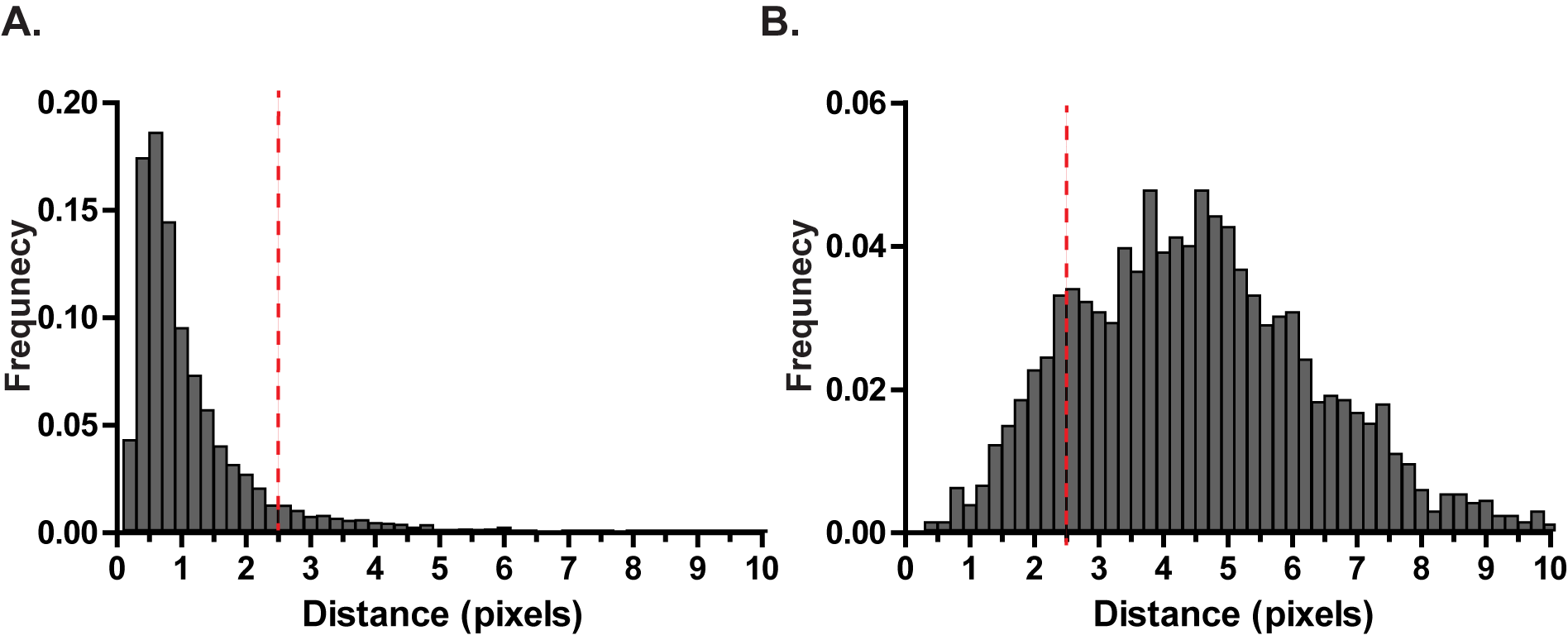

Supplement: Figure S2 — Empirical determination of the maximum distance threshold between the centers of spots which defines colocalization. (A) The distances between the centers of colocalized spots were empirically determined by using two probe sets targeting different regions of the same viral RNA. Two different regions of NA vRNA were bound by two probe sets, one labeled with Cy3 fluorophore and the other labeled with Cy5 fluorophore. Z-stack images were taken in both fluorescence channels and the centers for the Cy3 spots and Cy5 spots were localized in 3D space using the spot detection program. The distances between the Cy3 spots and their nearest-neighboring Cy5 spots were measured. The Distributions of the distances between the neighboring spots of different colors is shown here. The distances between the centers of the Cy3 and Cy5 spots were found mainly within 1 pixel (102 nm) and spread to approximately 2.5 pixels (255 nm). Since electron microscopy analysis suggested the packaged vRNPs range from 80 nm to 100 nm (the distances between Cy3 and Cy5 spots are theoretically within 1 pixel), the nearest neighbor distances measured here likely represent the accuracy of measurement of the same diffraction-limited object in Cy3 and Cy5 channels. We therefore define spots as being colocalized if their centers are within 2.5 pixels. (B) In contrast to colocalizing spots, the distribution of nearest neighbor distances between the NA vRNAs (Cy5 spots) and β-actin mRNA (Cy3 spots) in infected cells shows that the nearest-neighbor distances range from 1 pixel to 10 pixels (1020 nm), with most of the spots clustered within a range from 3.5 pixels to 6 pixels. The red dashed lines show the 2.5 pixels distance threshold to define colocalization of spots in the quantitative colocalization analysis. (TIF) [file ppat.1003358.s002.tif]

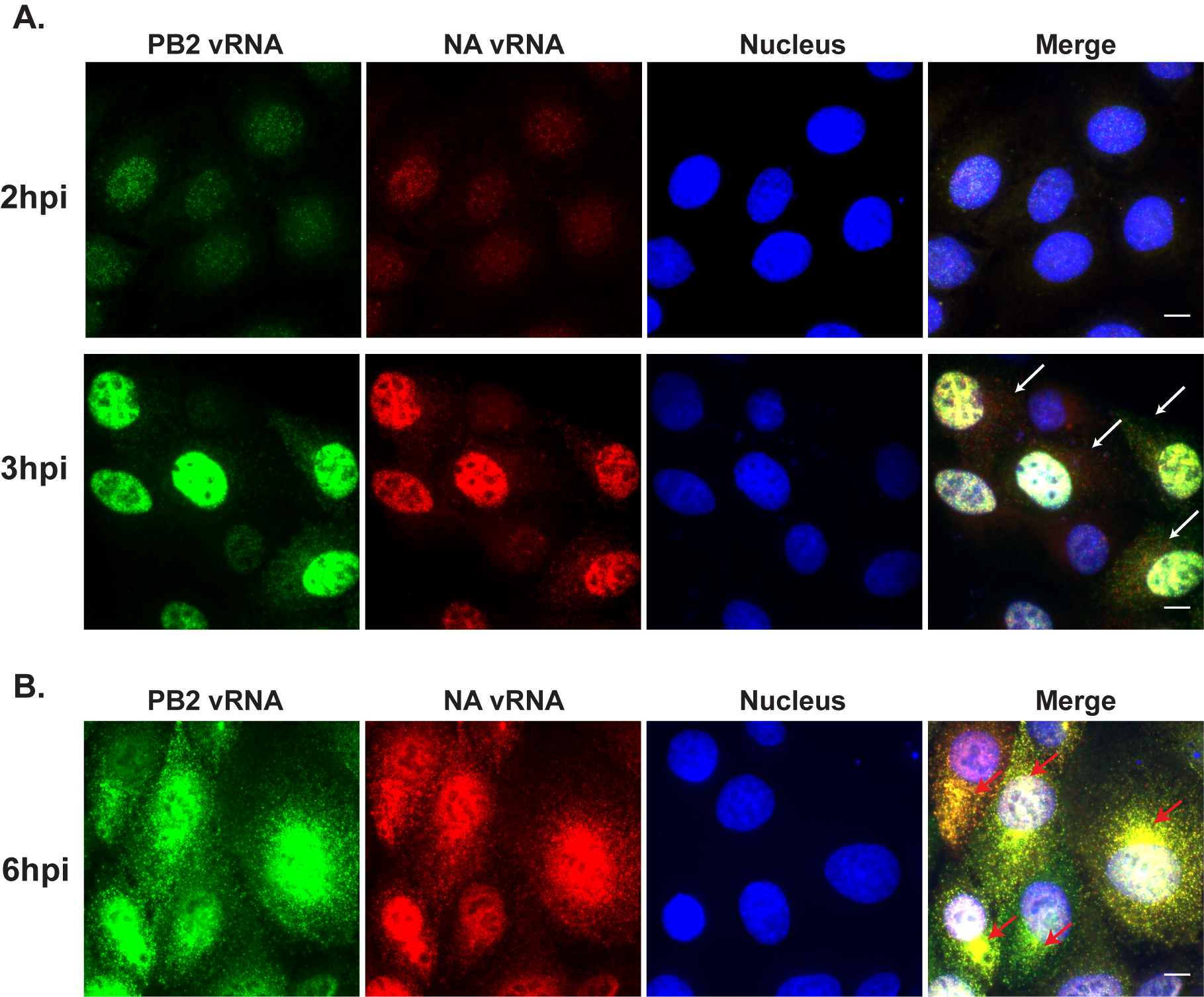

Supplement: Figure S3 — Distribution of vRNPs in the cytoplasm. Maximum intensity merge images of MDCK cells infected with PR8 virus at 2, 3 (A) and 6 (B) hpi. The cells were probed against PB2 vRNAs (green), NA vRNAs (red) and stained with DAPI (blue) to define the nuclear regions. The white arrows show the exported vRNAs detected in the cytoplasm. The red arrows indicate the peri-nuclear accumulations of vRNAs. Scale bar = 10 µm. (TIF) [file ppat.1003358.s003.tif]

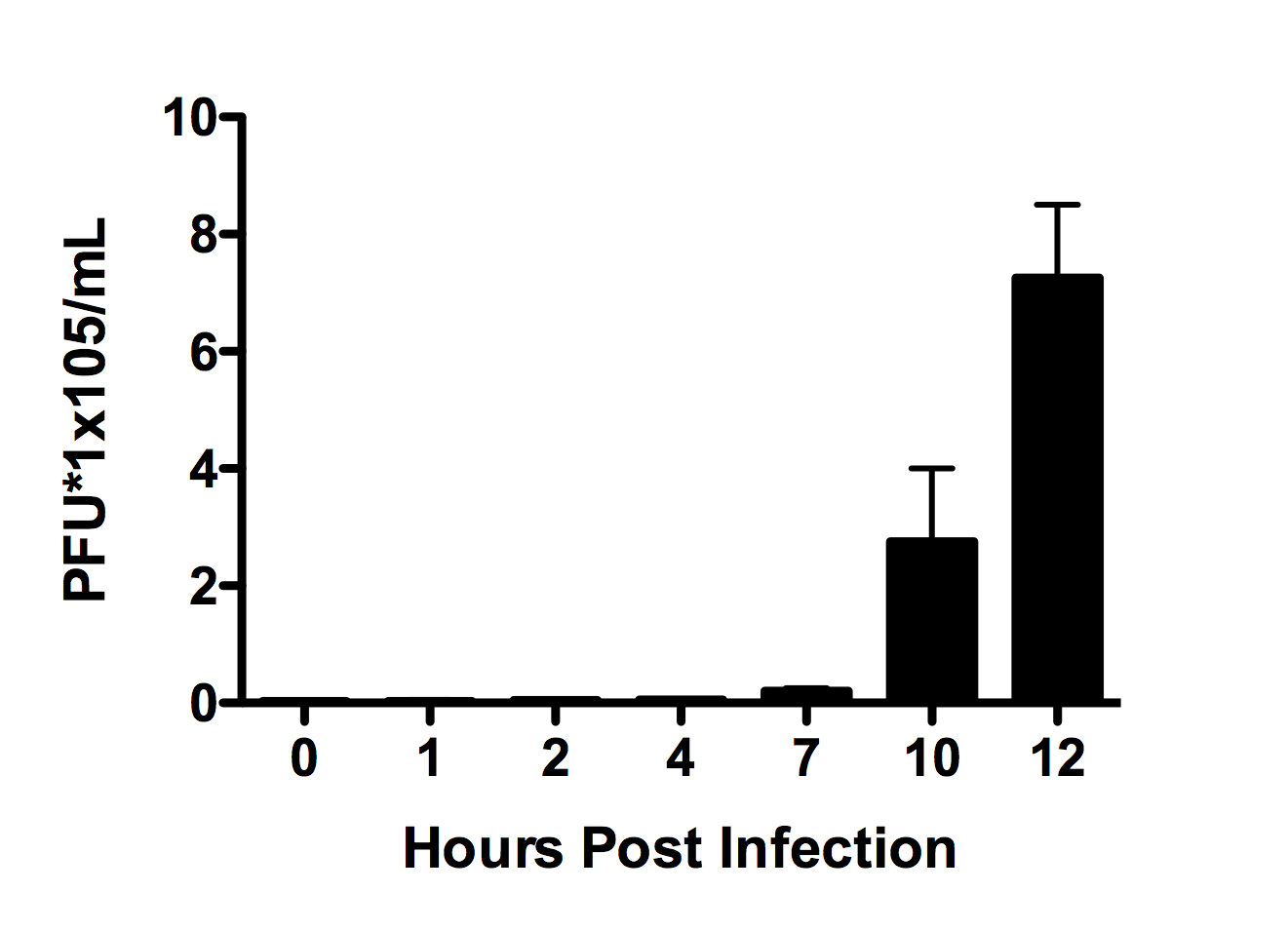

Supplement: Figure S4 — Growth kinetics of PR8 virus in MDCK cells. MDCK cells were infected with PR8 virus at MOI = 5. Supernatant of the infected MDCK cells were collected at 0, 1, 2, 4, 7, 10 and 12 hours post infection. The virus particles released into the supernatant were titered using standard plaque assays. The titer of the released virus particles at each time point is shown. (TIFF) [file ppat.1003358.s004.tif]

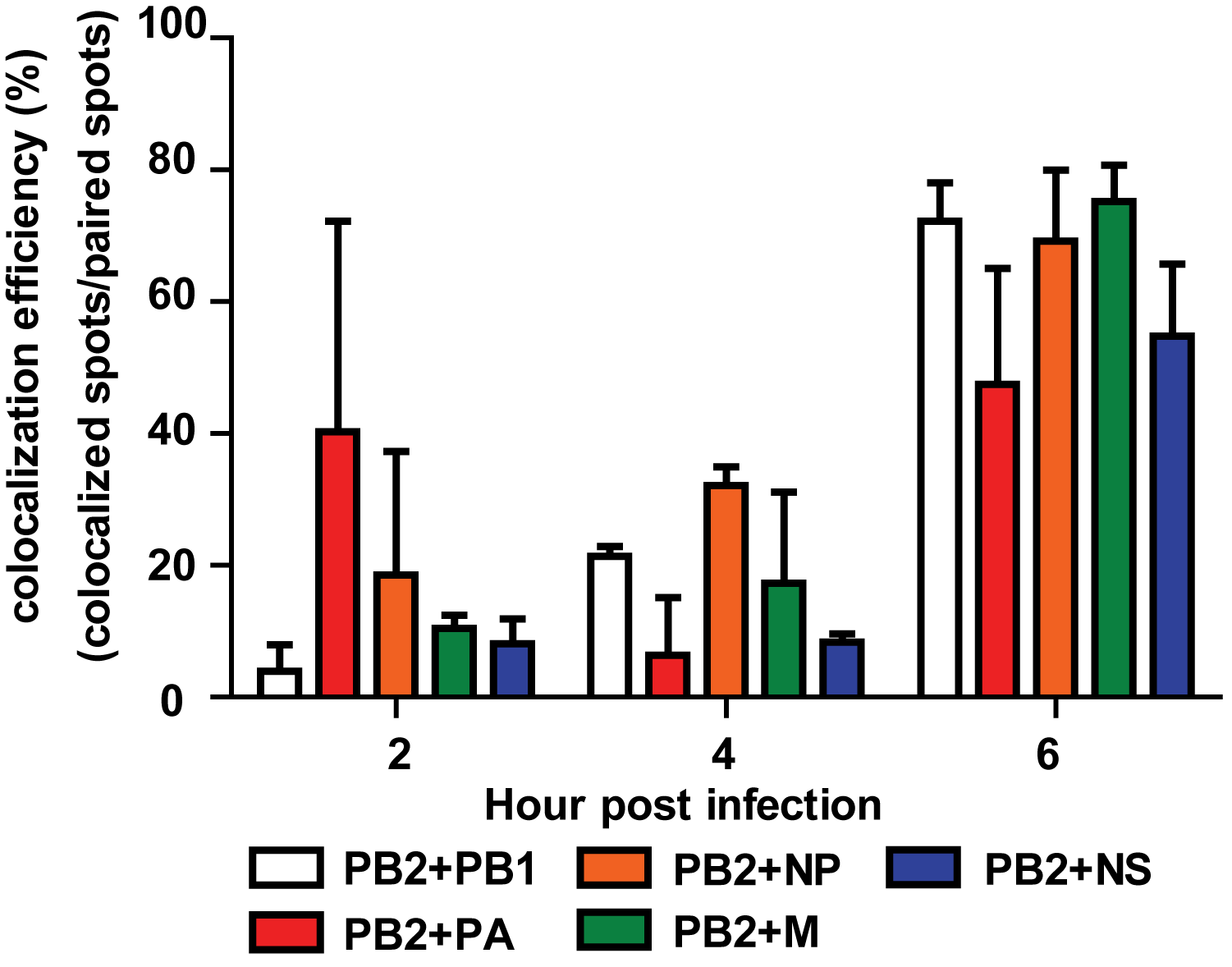

Supplement: Figure S5 — The colocalization of vRNPs of different identities can be observed at 6 hpi. MDCK cells were infected with PR8 virus at MOI = 5 and sm-FISH and colocalization analyses were performed for different pairs of vRNPs. Quantification of the colocalization efficiency between the indicated vRNA pairs is shown. Low localization efficiency is observed at 4 hpi for all the vRNA pairs tested while elevated colocalization efficiency of these vRNA pairs is detected at 6 hpi, demonstrating similar kinetics. (TIF) [file ppat.1003358.s005.tif]

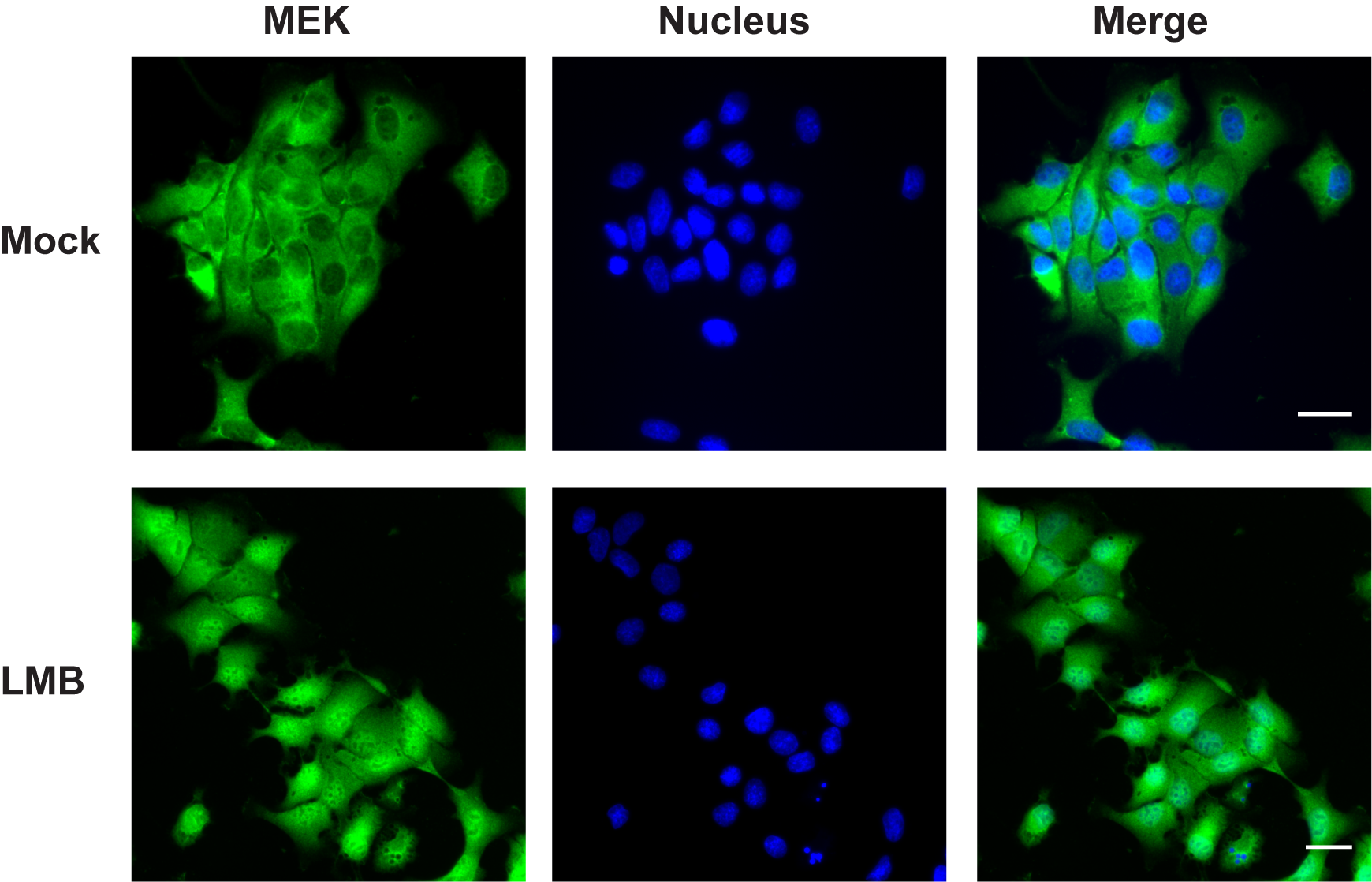

Supplement: Figure S6 — Positive control of LMB treatment on MDCK cells. As a positive control for the effect of LMB, we examined the nuclear-cytoplasmic shuttling protein MAP kinase (MEK) [44] in MDCK cells. The cells were first serum starved for 16 hours and incubated in infection media with 40 ng/ml of LMB for 1 hour (the same concentrations as virus infection was performed). The cells were then fixed with 4% formaldehyde for 10 min and immunofluorescence against the MEK protein using anti-MEK antibody (1∶100, Abcam). The fluorescence images of the MEK protein, the nuclei and the merged are shown. Nuclear accumulation of MEK in LMB treated cells reflects impaired export, whereas non treated cells display a clear cytoplasm localization pattern for MEK. Scale bar = 25 µm. (TIF) [file ppat.1003358.s006.tif]

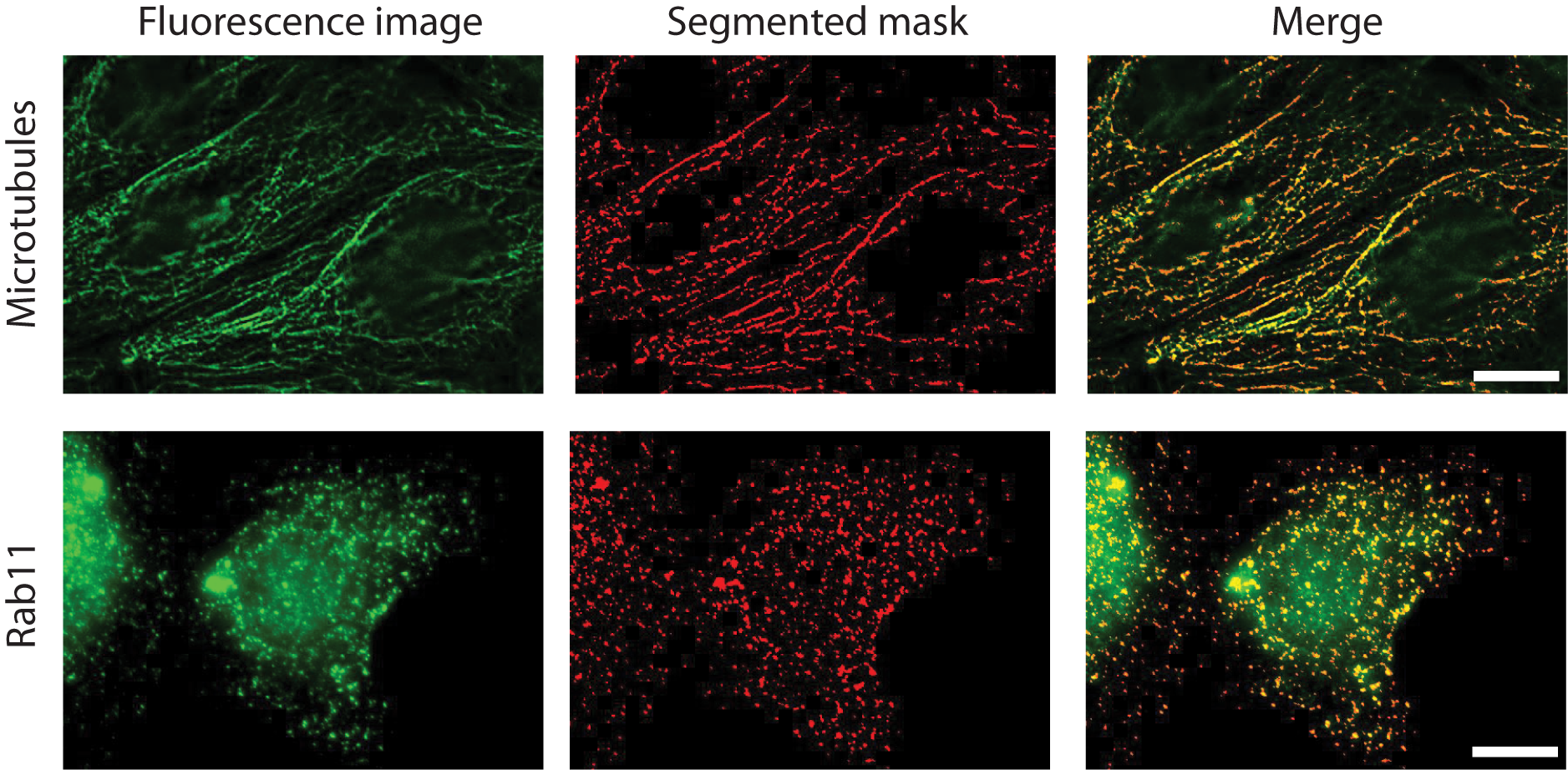

Supplement: Figure S7 — Automatic Segmentation of Microtubules and Rab11 Particles. Top: Microtubule Segmentation Example. Left, representative plane from a deconvolved image stack of microtubules labeled with an antibody against tubulin. Center, mask image showing the output of the automated segmentation algorithm for the plane on the left. Right, Overlay of data (green) and segmentation result (red). Bottom: Rab11 Particles Segmentation Example. Left, Maximum Intensity Projection of an image stack of Rab11 particles labeled with antibody against Rab11. Center, Maximum Intensity Projection of the output of the automated segmentation algorithm for the image stack represented on the left. Right, overlay of data (green) and segmentation results (red). Scale Bar: 10 µm. (TIF) [file ppat.1003358.s007.tif]

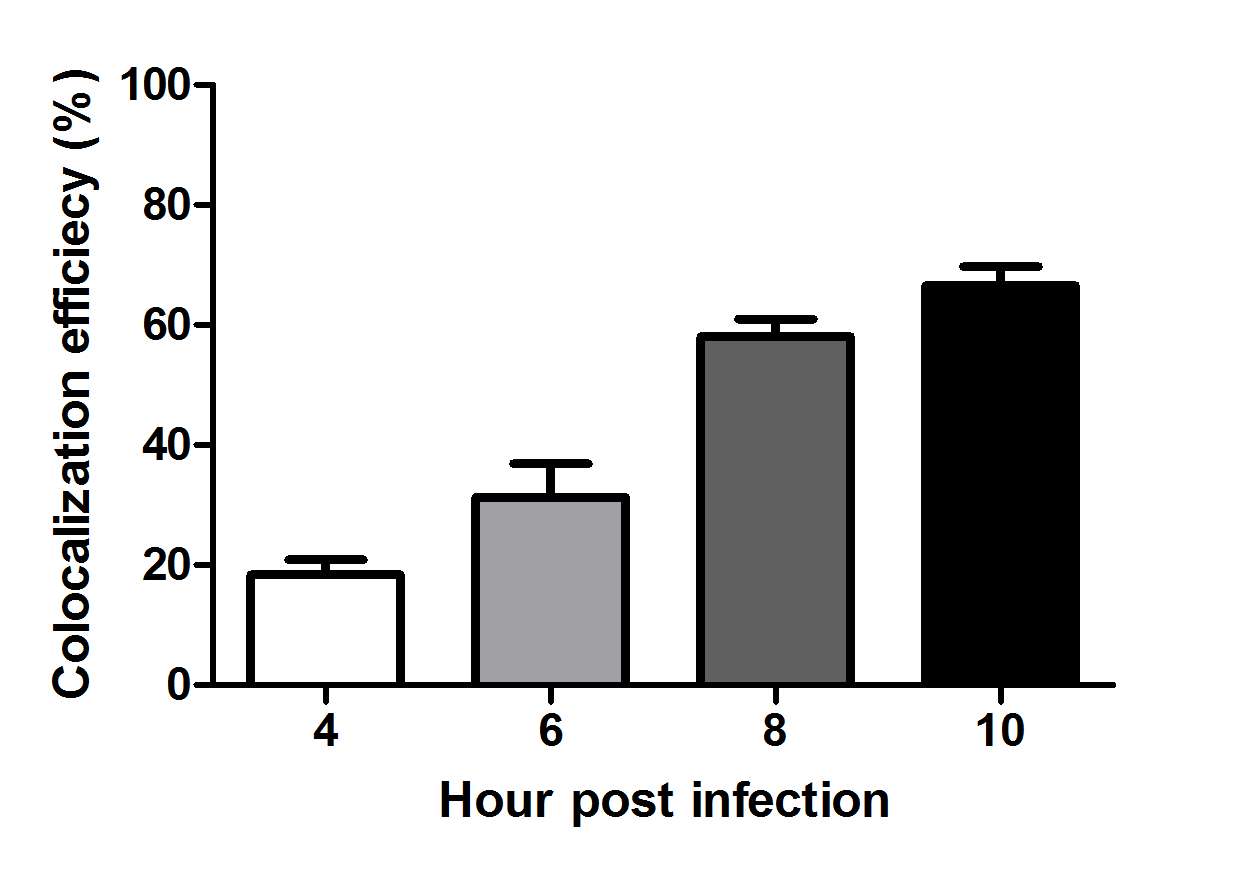

Supplement: Figure S8 — The colocalization of PB2 and NA vRNAs in A549 cells. A549 cells were infected with PR8 virus at MOI = 5 and sm-FISH and colocalization analyses were performed for PB2 and NA vRNAs (Cy5 labeled PB2 and Cy3 labeled NA vRNAs). Quantification of the colocalization efficiency between the two vRNAs is shown. Low localization efficiency is observed at 4 hpi and 6 hpi while elevated colocalization efficiency is detected at 8 hpi and 10 hpi, demonstrating a delayed kinetics compared to that in MDCK cells (Fig. 2C and Fig. 4B). (TIF) [file ppat.1003358.s008.tif]
