# Supplementary material for: Colocalization of Different Influenza Viral RNA Segments in the Cytoplasm before Viral Budding as Shown by Single-molecule Sensitivity FISH Analysis
Source: PLoS Pathog. 2013 May 9;9(5):e1003358. doi: 10.1371/journal.ppat.1003358 (PMC3649991; doi:10.1371/journal.ppat.1003358)
Supplement: Text S1 — Probes sequences used to target vRNA or cellular mRNA in influenza virus infected cells. Sequences for each probe used to target influenza vRNA or cellular mRNA are listed here. (DOCX) [file ppat.1003358.s009.docx]

**Supplementary Material**

**Supplementary Note 1**

**Probes sequences used to target vRNA or cellular mRNA in influenza virus infected cells.**

| RNA target | Probe sequence | RNA target | Probe sequence |
| --- | --- | --- | --- |
| PB2 vRNA | | NA vRNA | |
| PR8-PB2-vRNA-155-2194_1 | aactgagcaaccttgcgaaa | PR8-NA-vRNA-24-1393_1 | cagcattgacaagtagtctg |
| PR8-PB2-vRNA-155-2194_2 | aagacaagagatatgggcca | PR8-NA-vRNA-24-1393_2 | tagattggtcttggccagac |
| PR8-PB2-vRNA-155-2194_3 | ttctgaggggattcctcatt | PR8-NA-vRNA-24-1393_3 | tgtggcgtgaatagtgatac |
| PR8-PB2-vRNA-155-2194_4 | aactgaagacccagatgaag | PR8-NA-vRNA-24-1393_4 | tagtgcgagcagcatttctt |
| PR8-PB2-vRNA-155-2194_5 | tcacagttctcggaaaggat | PR8-NA-vRNA-24-1393_5 | ggggacgacctaaagaaaaa |
| PR8-PB2-vRNA-155-2194_6 | tataacaaggccacgaagag | PR8-NA-vRNA-24-1393_6 | cgtgcttctgggttgaatta |
| PR8-PB2-vRNA-155-2194_7 | gggcaattctcctgtattca | PR8-NA-vRNA-24-1393_7 | aacagggctagactgtatga |
| PR8-PB2-vRNA-155-2194_8 | gggatcaggaatgagaatac | PR8-NA-vRNA-24-1393_8 | gtttcgttcaacatcctgag |
| PR8-PB2-vRNA-155-2194_9 | gcagttctcctcatttactg | PR8-NA-vRNA-24-1393_9 | actgattggtcagggtatag |
| PR8-PB2-vRNA-155-2194_10 | ctccaccaaagcaaagtaga | PR8-NA-vRNA-24-1393_10 | taggcaagatgttgtggcaa |
| PR8-PB2-vRNA-155-2194_11 | aaaacttcttcccttcgcag | PR8-NA-vRNA-24-1393_11 | gagactgatagtaagttctc |
| PR8-PB2-vRNA-155-2194_12 | gacatttgataccgcacaga | PR8-NA-vRNA-24-1393_12 | ttgggatcctaatggatgga |
| PR8-PB2-vRNA-155-2194_13 | caacaaatgagggatgtgct | PR8-NA-vRNA-24-1393_13 | tccagacatgggtttgagat |
| PR8-PB2-vRNA-155-2194_14 | gtgggtttgtaagaactctg | PR8-NA-vRNA-24-1393_14 | aggaaggaccaaaagtcaca |
| PR8-PB2-vRNA-155-2194_15 | aaggccattagaggccaata | PR8-NA-vRNA-24-1393_15 | ggtatggtaatggtgtttgg |
| PR8-PB2-vRNA-155-2194_16 | ccagaaccctacaatgctat | PR8-NA-vRNA-24-1393_16 | gcaaacggagtaaagggatt |
| PR8-PB2-vRNA-155-2194_17 | catcagaaactgggaaactg | PR8-NA-vRNA-24-1393_17 | tggtccagtgtatgttgatg |
| PR8-PB2-vRNA-155-2194_18 | cctgaatcagtgttggtcaa | PR8-NA-vRNA-24-1393_18 | tacatctgcagtggggtttt |
| PR8-PB2-vRNA-155-2194_19 | cgtcaatgatgtgggagatt | PR8-NA-vRNA-24-1393_19 | gtctttcgatcaaaacctgg |
| PR8-PB2-vRNA-155-2194_20 | gggaacagagaaactgacaa | PR8-NA-vRNA-24-1393_20 | acaactggcatggttcgaac |
| PR8-PB2-vRNA-155-2194_21 | aatgtactactgtctcccga | PR8-NA-vRNA-24-1393_21 | aaagtgatgtgtgtgtgcag |
| PR8-PB2-vRNA-155-2194_22 | ttttgagaatccgggaccaa | PR8-NA-vRNA-24-1393_22 | tgttcctgttaccctgatac |
| PR8-PB2-vRNA-155-2194_23 | tgtagatgagtactccagca | PR8-NA-vRNA-24-1393_23 | acctaattctcactatgagg |
| PR8-PB2-vRNA-155-2194_24 | gaggagtgagaatcagcaaa | PR8-NA-vRNA-24-1393_24 | cgaaaaggggaaggttacta |
| PR8-PB2-vRNA-155-2194_25 | ccaagcatcgagatgtcaat | PR8-NA-vRNA-24-1393_25 | gctggcctcgtacaaaattt |
| PR8-PB2-vRNA-155-2194_26 | tgggatattgccagacatga | PR8-NA-vRNA-24-1393_26 | tataatgactgatggcccga |
| PR8-PB2-vRNA-155-2194_27 | tcgacaatgtgatgggaatg | PR8-NA-vRNA-24-1393_27 | gcctgtgtaaatggttcatg |
| PR8-PB2-vRNA-155-2194_28 | caaaattggggagttgaacc | PR8-NA-vRNA-24-1393_28 | gaggacacaagagtctgaat |
| PR8-PB2-vRNA-155-2194_29 | gaaggatgcgaaagtgcttt | PR8-NA-vRNA-24-1393_29 | ccataaaaagttggaggaag |
| PR8-PB2-vRNA-155-2194_30 | gcgattgaatcctatgcatc | PR8-NA-vRNA-24-1393_30 | ggagcagtggctgtattaaa |
| PR8-PB2-vRNA-155-2194_31 | gaatttcgtcaatagggcga | PR8-NA-vRNA-24-1393_31 | cggaatttcaggtccagata |
| PR8-PB2-vRNA-155-2194_32 | gataaaagcagtcagaggtg | PR8-NA-vRNA-24-1393_32 | agtgcatgtcatgatggcat |
| PR8-PB2-vRNA-155-2194_33 | ggccatggtattttcacaag | PR8-NA-vRNA-24-1393_33 | ttgaatcggttgcttggtca |
| PR8-PB2-vRNA-155-2194_34 | gtcgattgccgaagcaataa | PR8-NA-vRNA-24-1393_34 | tccgtccccgtacaattcaa |
| PR8-PB2-vRNA-155-2194_35 | attcagctgatagtgagtgg | PR8-NA-vRNA-24-1393_35 | tatagggccttaatgagctg |
| PR8-PB2-vRNA-155-2194_36 | cagaaaagcaaccaggagat | PR8-NA-vRNA-24-1393_36 | tgggactgttaaggacagaa |
| PR8-PB2-vRNA-155-2194_37 | aatggttgggagaagagcaa | PR8-NA-vRNA-24-1393_37 | gccttactgaatgacaagca |
| PR8-PB2-vRNA-155-2194_38 | gcatgagggatatgaagagt | PR8-NA-vRNA-24-1393_38 | gaccttttttctgacccaag |
| PR8-PB2-vRNA-155-2194_39 | cgggcaatcttcaaacattg | PR8-NA-vRNA-24-1393_39 | catgttctcacttggaatgc |
| PR8-PB2-vRNA-155-2194_40 | tcagtcaagagagaggaaga | PR8-NA-vRNA-24-1393_40 | ccaaaggagacgtttttgtc |
| PR8-PB2-vRNA-155-2194_41 | ccttcagttttggtggattc | PR8-NA-vRNA-24-1393_41 | gggctatatacagcaaagac |
| PR8-PB2-vRNA-155-2194_42 | atgggactgagaattagctc | PR8-NA-vRNA-24-1393_42 | ttcatctctttgtcccatcc |
| PR8-PB2-vRNA-155-2194_43 | aagccgtggatatatgcaag | PR8-NA-vRNA-24-1393_43 | cttcagtgatattaaccggc |
| PR8-PB2-vRNA-155-2194_44 | ttaggcagaacccaacagaa | PR8-NA-vRNA-24-1393_44 | aatagcacctgggtaaagga |
| PR8-PB2-vRNA-155-2194_45 | gtggaattaggatggtagac | PR8-NA-vRNA-24-1393_45 | gcaaccaaaacatcattacc |
| PR8-PB2-vRNA-155-2194_46 | ttattggagatgtgccacag | PR8-NA-vRNA-24-1393_46 | ggaagtcaaaaccatactgg |
| PR8-PB2-vRNA-155-2194_47 | atcagcagatccactagcat | PR8-NA-vRNA-24-1393_47 | gcctaatattgcaaataggg |
| PR8-PB2-vRNA-155-2194_48 | acatagtgagaagagctgca | PR8-NA-vRNA-24-1393_48 | tgtctggtagtcggactaat |
| PB1 vRNA | | PA vRNA | |
| PR8-PB1-vRNA-164-2257_1 | ttcatacagaagaccagtcg | PR8-PA-vRNA-1 | cttcttggttcaactccttc |
| PR8-PB1-vRNA-164-2257_2 | gtaccaaaggtgctgcaatt | PR8-PA-vRNA-2 | aatgatccctgggttttgct |
| PR8-PB1-vRNA-164-2257_3 | ccccaaaagaaatcgatcca | PR8-PA-vRNA-3 | atgaagcaattgaggagtgc |
| PR8-PB1-vRNA-164-2257_4 | tgttgcaacaacacactcct | PR8-PA-vRNA-4 | aacctgggacctttgatctt |
| PR8-PB1-vRNA-164-2257_5 | ccagccaaaaacatggagta | PR8-PA-vRNA-5 | ttatcgttcaggctcttagg |
| PR8-PB1-vRNA-164-2257_6 | acaatgcagtgatgatgcca | PR8-PA-vRNA-6 | catctccacaactagaagga |
| PR8-PB1-vRNA-164-2257_7 | cccatttgtcagccataaag | PR8-PA-vRNA-7 | caaagtcggtattcaacagc |
| PR8-PB1-vRNA-164-2257_8 | tgatggatgaggattaccag | PR8-PA-vRNA-8 | aagttccattgggaaggtct |
| PR8-PB1-vRNA-164-2257_9 | gaagtctgcctaaaatggga | PR8-PA-vRNA-9 | atcagaaacatggcccattg |
| PR8-PB1-vRNA-164-2257_10 | cggaggcccaaatttataca | PR8-PA-vRNA-10 | cctctgtcaaagagaaagac |
| PR8-PB1-vRNA-164-2257_11 | aagaaactgtgggagcaaac | PR8-PA-vRNA-11 | cctccagtcacttcaacaaa |
| PR8-PB1-vRNA-164-2257_12 | gacacacaaatacaaacccg | PR8-PA-vRNA-12 | aaatggggaatggagatgag |
| PR8-PB1-vRNA-164-2257_13 | ttacaggtacacgtaccgat | PR8-PA-vRNA-13 | gtgagaacaaatggaacctc |
| PR8-PB1-vRNA-164-2257_14 | gcccttcagttgttcatcaa | PR8-PA-vRNA-14 | ataagaagtgccataggcca |
| PR8-PB1-vRNA-164-2257_15 | atgatcttggtccagcaaca | PR8-PA-vRNA-15 | ctgtgttcttgagataggag |
| PR8-PB1-vRNA-164-2257_16 | cggacatgagtattggagtt | PR8-PA-vRNA-16 | cccaagacttgaaccacata |
| PR8-PB1-vRNA-164-2257_17 | ttgccaatttcagcatggag | PR8-PA-vRNA-17 | tgagcatggagttttctctc |
| PR8-PB1-vRNA-164-2257_18 | cctgtaagctacttggaatc | PR8-PA-vRNA-18 | aatgacaccgacgtggtaaa |
| PR8-PB1-vRNA-164-2257_19 | atcatgaagggattcaagcc | PR8-PA-vRNA-19 | cgaaagaccaacttgtatgg |
| PR8-PB1-vRNA-164-2257_20 | tgctctgattgtgaatgcac | PR8-PA-vRNA-20 | caagtgtagaactaaggagg |
| PR8-PB1-vRNA-164-2257_21 | gtcttcaatcctctgacgat | PR8-PA-vRNA-21 | gcagcaatggatgatttcca |
| PR8-PB1-vRNA-164-2257_22 | aagactacttactggtggga | PR8-PA-vRNA-22 | gtacatcaatactgccttgc |
| PR8-PB1-vRNA-164-2257_23 | ccatcctgaatcttggacaa | PR8-PA-vRNA-23 | gcagagccacagaatacata |
| PR8-PB1-vRNA-164-2257_24 | gttaagcactgtattaggcg | PR8-PA-vRNA-24 | cattgcaagcatgagaagga |
| PR8-PB1-vRNA-164-2257_25 | gaatgatgatgggcatgttc | PR8-PA-vRNA-25 | gagaagatgtggctccaatt |
| PR8-PB1-vRNA-164-2257_26 | atagaggggactgcatcatt | PR8-PA-vRNA-26 | attcaagctggatagagctc |
| PR8-PB1-vRNA-164-2257_27 | tgaaaaaatccgaccgctct | PR8-PA-vRNA-27 | gtttaacaaggcatgcgaac |
| PR8-PB1-vRNA-164-2257_28 | gctagcaagcatcgatttga | PR8-PA-vRNA-28 | tcgctagcaagttggattca |
| PR8-PB1-vRNA-164-2257_29 | gactgggaaaagggtatatg | PR8-PA-vRNA-29 | cgactgtaaagatgtaggtg |
| PR8-PB1-vRNA-164-2257_30 | cccgaatggttcagaaatgt | PR8-PA-vRNA-30 | atggcaccagaaaaggtaga |
| PR8-PB1-vRNA-164-2257_31 | ggatgtttttggccatgatc | PR8-PA-vRNA-31 | aacaagtcagctaaagtggg |
| PR8-PB1-vRNA-164-2257_32 | ctggagataacaccaaatgg | PR8-PA-vRNA-32 | ggacattgagaatgaggaga |
| PR8-PB1-vRNA-164-2257_33 | caccgaactttctttcacca | PR8-PA-vRNA-33 | tgtcatggaagcaagtactg |
| PR8-PB1-vRNA-164-2257_34 | gagaagaaagcaaagttggc | PR8-PA-vRNA-34 | gttaaaccacacgaaaaggg |
| PR8-PB1-vRNA-164-2257_35 | aacaatcagggttgccagtt | PR8-PA-vRNA-35 | ttctttggatggaaggaacc |
| PR8-PB1-vRNA-164-2257_36 | ggcaaggagtatatgtgaga | PR8-PA-vRNA-36 | ggaataccgctatatgatgc |
| PR8-PB1-vRNA-164-2257_37 | gatgcaaataagggggtttg | PR8-PA-vRNA-37 | ttgaggacccaagtcatgaa |
| PR8-PB1-vRNA-164-2257_38 | aaacggagagcaattgcaac | PR8-PA-vRNA-38 | ctgctgatggatgccttaaa |
| PR8-PB1-vRNA-164-2257_39 | aaagatgctgagagagggaa | PR8-PA-vRNA-39 | accacttagacttccgaatg |
| PR8-PB1-vRNA-164-2257_40 | cattgaccctgaacacaatg | PR8-PA-vRNA-40 | gctgtctcaaatgtccaaag |
| PR8-PB1-vRNA-164-2257_41 | gggtgagagacaatatgact | PR8-PA-vRNA-41 | tatgtggatggattcgaacc |
| PR8-PB1-vRNA-164-2257_42 | ggggatcacaactcattttc | PR8-PA-vRNA-42 | gaacttctccagccttgaaa |
| PR8-PB1-vRNA-164-2257_43 | gctcatagacttccttaagg | PR8-PA-vRNA-43 | atcacaggaacaatgcgcaa |
| PR8-PB1-vRNA-164-2257_44 | ttcagatcaaatggcctcac | PR8-PA-vRNA-44 | gagaggagaagagacaattg |
| PR8-PB1-vRNA-164-2257_45 | gcattggccaacacaataga | PR8-PA-vRNA-45 | aagacaagaaatggccagca |
| PR8-PB1-vRNA-164-2257_46 | tagaaaccaacctgctgcaa | PR8-PA-vRNA-46 | gctaggatcaaaaccagact |
| PR8-PB1-vRNA-164-2257_47 | agacctatgactggactcta | PR8-PA-vRNA-47 | gactacactctcgatgaaga |
| PR8-PB1-vRNA-164-2257_48 | agtagacaagctgacacaag | PR8-PA-vRNA-48 | tcgttcactggggaagaaat |
| HA vRNA (HA1 subtype) | | HA9 vRNA (HA9 subtype) | |
| PR8-HA-vRNA-23 | ctcaaacgcatcaatgcatg | PR8cH9-HA-vRNA-177-854-1 | gccatggaagaatcctgaag |
| PR8-HA-vRNA-24 | atgctttcgcactgagtaga | PR8cH9-HA-vRNA-177-854-2 | atggacacgttctttcagga |
| PR8-HA-vRNA-25 | ctaaaacccggagacacaat | PR8cH9-HA-vRNA-177-854-3 | gggaatctaatcgctccatg |
| PR8-HA-vRNA-26 | ggaggatgaactattactgg | PR8cH9-HA-vRNA-177-854-4 | aacattgcgagtaagatcca |
| PR8-HA-vRNA-27 | gcagaaagacccaaagtaag | PR8cH9-HA-vRNA-177-854-5 | ttggtcggtactaaaaccag |
| PR8-HA-vRNA-28 | ataacaggagatttaccccg | PR8cH9-HA-vRNA-177-854-6 | caatggtctgcagggaagaa |
| PR8-HA-vRNA-29 | gcttatgtctctgtagtgac | PR8cH9-HA-vRNA-177-854-7 | tgatagggccaaggcccctt |
| PR8-HA-vRNA-30 | gcctaacagtaaggaacaac | PR8cH9-HA-vRNA-177-854-8 | ttgaataggaccttcaaacc |
| PR8-HA-vRNA-31 | cttgtactgtggggtattca | PR8cH9-HA-vRNA-177-854-9 | aacaagcgtgacaacagaag |
| PR8-HA-vRNA-32 | gctcatacccaaagctgaaa | PR8cH9-HA-vRNA-177-854-10 | tgtacataagaaacgacaca |
| PR8-HA-vRNA-33 | aatttgctatggctgacgga | PR8cH9-HA-vRNA-177-854-11 | agagcattcttttcgtgtgg |
| PR8-HA-vRNA-34 | catgaggggaaaagcagttt | PR8cH9-HA-vRNA-177-854-12 | caatacacaaataacagggg |
| PR8-HA-vRNA-35 | acaacacaaacggagtaacg | PR8cH9-HA-vRNA-177-854-13 | cggtttttaccctgttcaag |
| PR8-HA-vRNA-36 | ttcccaaagaaagctcatgg | PR8cH9-HA-vRNA-177-854-14 | tgagatggctgactcaaaag |
| PR8-HA-vRNA-37 | gctcagtgtcatcattcgaa | PR8cH9-HA-vRNA-177-854-15 | tcaggttcattctacaggag |
| PR8-HA-vRNA-38 | ttcatcgactatgaggagct | PR8cH9-HA-vRNA-177-854-16 | ttacactggaacaagcagag |
| PR8-HA-vRNA-39 | caccaaactctgagaatgga | PR8cH9-HA-vRNA-177-854-17 | cagacacaacctggaatgtg |
| PR8-HA-vRNA-40 | gagatcatggtcctacattg | PR8cH9-HA-vRNA-177-854-18 | cgctagttcctaccaaagaa |
| PR8-HA-vRNA-41 | aaatgtaacatcgccggatg | PR8cH9-HA-vRNA-177-854-19 | agaggaactcaggacacttt |
| PR8-HA-vRNA-42 | aggaatagccccactacaat | PR8cH9-HA-vRNA-177-854-20 | accctgggaatgtagaaaac |
| PR8-HA-vRNA-43 | gccacaacggaaaactatgt | PR8cH9-HA-vRNA-177-854-21 | tcagctgtaaatggaacgtg |
|  |  | PR8cH9-HA-vRNA-177-854-22 | atggtcctacatcgtcgaaa |
|  |  | PR8cH9-HA-vRNA-177-854-23 | acctgctgttagaaggaaga |
|  |  | PR8cH9-HA-vRNA-177-854-24 | gtctatggcaacccttcttg |
|  |  | PR8cH9-HA-vRNA-177-854-25 | acacatgcactattgaagga |
| NP vRNA | | M vRNA | |
| PR8-NP-vRNA-1 | gacatgaggaccgaaatcat | PR8-M-vRNA-1 | tgtggattcttgatcgtctt |
| PR8-NP-vRNA-2 | ggaatacagagggaagaaca | PR8-M-vRNA-2 | attgggatcttgcacttgac |
| PR8-NP-vRNA-3 | caaccattatggcagcattc | PR8-M-vRNA-3 | tctcgctattgccgcaaata |
| PR8-NP-vRNA-4 | cagtacagagaaatctccct | PR8-M-vRNA-4 | gatgcaacggttcaagtgat |
| PR8-NP-vRNA-5 | gccaaatcagcatacaacct | PR8-M-vRNA-5 | atcagaaacgaatgggggtg |
| PR8-NP-vRNA-6 | aaacaccaatcaacagaggg | PR8-M-vRNA-6 | cttcttgaaaatttgcaggc |
| PR8-NP-vRNA-7 | tgaactgagaagcaggtact | PR8-M-vRNA-7 | cagtgctggtctgaaaaatg |
| PR8-NP-vRNA-8 | gaggagttcaaattgcttcc | PR8-M-vRNA-8 | accattgggactcatcctag |
| PR8-NP-vRNA-9 | ttcatcaaagggacgaaggt | PR8-M-vRNA-9 | acaaatggtgcaagcgatga |
| PR8-NP-vRNA-10 | gccgcatttgaagatctaag | PR8-M-vRNA-10 | tggaggttgctagtcaggct |
| PR8-NP-vRNA-11 | aagagtcaactggtgtggat | PR8-M-vRNA-11 | agtgagcaagcagcagaggc |
| PR8-NP-vRNA-12 | accaaatgagaatccagcac | PR8-M-vRNA-12 | tatggagcaaatggctggat |
| PR8-NP-vRNA-13 | agccaagtgtacagcctaat | PR8-M-vRNA-13 | tagccagcactacagctaag |
| PR8-NP-vRNA-14 | ccctttcagactgcttcaaa | PR8-M-vRNA-14 | agacatgagaacagaatggt |
| PR8-NP-vRNA-15 | ggatactctctagtcggaat | PR8-M-vRNA-15 | gacaacaaccaatccactaa |
| PR8-NP-vRNA-16 | tgggtacgactttgaaagag | PR8-M-vRNA-16 | atcggtctcataggcaaatg |
| PR8-NP-vRNA-17 | tcatattgagagggtcggtt | PR8-M-vRNA-17 | gaacagattgctgactccca |
| PR8-NP-vRNA-18 | tcacttttctagcacggtct | PR8-M-vRNA-18 | tggcctggtatgtgcaacct |
| PR8-NP-vRNA-19 | atggatcaagtgagagagag | PR8-M-vRNA-19 | ctgtgaccactgaagtggca |
| PR8-NP-vRNA-20 | caaactgctgcacaaaaagc | PR8-M-vRNA-20 | ctcatatacaacaggatggg |
| PR8-NP-vRNA-21 | gtgcaacattctcaaaggga | PR8-M-vRNA-21 | tgcacttgccagttgtatgg |
| PR8-NP-vRNA-22 | ggtgagaatggacgaaaaac | PR8-M-vRNA-22 | tctcactcagttattctgct |
| PR8-NP-vRNA-23 | caatgatcggaacttctgga | PR8-M-vRNA-23 | taacattccatggggccaaa |
| PR8-NP-vRNA-24 | tggtcaggatgatcaaacgt | PR8-M-vRNA-24 | tataggaagctcaagaggga |
| PR8-NP-vRNA-25 | gttggaacaatggtgatgga | PR8-M-vRNA-25 | catggacaaagcagttaaac |
| PR8-NP-vRNA-26 | tgatgcaaggttcaactctc | PR8-M-vRNA-26 | atgggaacggggatccaaat |
| PR8-NP-vRNA-27 | agaggacaagagctcttgtt | PR8-M-vRNA-27 | cgctttgtccaaaatgccct |
| PR8-NP-vRNA-28 | catgatgatctggcattcca | PR8-M-vRNA-28 | tgagcgaggactgcagcgta |
| PR8-NP-vRNA-29 | ataatggtgacgatgcaacg | PR8-M-vRNA-29 | tttgtgttcacgctcaccgt |
| PR8-NP-vRNA-30 | agaaataaggcgaatctggc | PR8-M-vRNA-30 | tctgactaaggggattttag |
| PR8-NP-vRNA-31 | ggatgagagaactcatcctt | PR8-M-vRNA-31 | agacaagaccaatcctgtca |
| PR8-NP-vRNA-32 | ggacctatatacaggagagt | PR8-M-vRNA-32 | ttgaggttctcatggaatgg |
| PR8-NP-vRNA-33 | cggggaaagatcctaagaaa |  |  |
| PR8-NP-vRNA-34 | atacctggaagaacatccca |  |  |
| PR8-NP-vRNA-35 | ctgcttttgacgaaaggaga |  |  |
| PR8-NP-vRNA-36 | cggttgatccaaaacagctt |  |  |
| PR8-NP-vRNA-37 | ccgaactcaaactcagtgat |  |  |
| PR8-NP-vRNA-38 | gtggaattggacgattctac |  |  |
| PR8-NP-vRNA-39 | gcatccgtcggaaaaatgat |  |  |
| PR8-NP-vRNA-40 | ccagaatgccactgaaatca |  |  |
| Canine beta actin mRNA | | NS vRNA | |
| Dog-β-actin-mRNA-1 | ttctccatgtcgtcccagtt | PR8-NS-vRNA-1 | gcaagccttacatctattgc |
| Dog-β-actin-mRNA-2 | ttgtagaaggtgtggtgcca | PR8-NS-vRNA-2 | cagagaatagttttgagcaa |
| Dog-β-actin-mRNA-3 | atcttctcacggttggcttt | PR8-NS-vRNA-3 | gtgagacacaaactgaagat |
| Dog-β-actin-mRNA-4 | acgtctcgaacatgatctga | PR8-NS-vRNA-4 | gggaacaattaggtcagaag |
| Dog-β-actin-mRNA-5 | acatacatggctggggtgtt | PR8-NS-vRNA-5 | cactccaaaacagaaacgag |
| Dog-β-actin-mRNA-6 | tccatgacaataccagtggt | PR8-NS-vRNA-6 | taatgagaatgggagacctc |
| Dog-β-actin-mRNA-7 | ttcatgaggtagtcagtcag | PR8-NS-vRNA-7 | acagagattcgcttggagaa |
| Dog-β-actin-mRNA-8 | ttcttccttgatgtcacgca | PR8-NS-vRNA-8 | cagttcgagtctctgaaact |
| Dog-β-actin-mRNA-9 | tagttcgtagctcttctcca | PR8-NS-vRNA-9 | ggggacttgaatggaatgat |
| Dog-β-actin-mRNA-10 | ttgccaatggtgatcacctg | PR8-NS-vRNA-10 | aatgcagttggagtcctcat |
| Dog-β-actin-mRNA-11 | aggttggaagagtgcttttg | PR8-NS-vRNA-11 | acatactgctgaggatgtca |
| Dog-β-actin-mRNA-12 | atgattccatccccaggaaa | PR8-NS-vRNA-12 | caccattgccttctcttcca |
| Dog-β-actin-mRNA-13 | tgaaggtggtctcgtggata | PR8-NS-vRNA-13 | ggagcaattgttggcgaaat |
| Dog-β-actin-mRNA-14 | acgtcgcacttcatgatgga | PR8-NS-vRNA-14 | tattgctaagggctttcacc |
| Dog-β-actin-mRNA-15 | atacaggtccttacggatgt | PR8-NS-vRNA-15 | tttttgaccggctggagact |
| Dog-β-actin-mRNA-16 | tacatggtggtacctccaga | PR8-NS-vRNA-16 | ctgaaagcgaacttcagtgt |
| Dog-β-actin-mRNA-17 | ttctgcatcctgtcagcaat | PR8-NS-vRNA-17 | ggcgatcatggataagaaca |
| Dog-β-actin-mRNA-18 | ttgatcttcatccttgctgg | PR8-NS-vRNA-18 | ccctctttgtatcagaatgg |
| Dog-β-actin-mRNA-19 | acacagaatacttgcgttcc | PR8-NS-vRNA-19 | tcatacccaagcagaaagtg |
| Dog-β-actin-mRNA-20 | atccacatctgctggaaggt | PR8-NS-vRNA-20 | atgtcaagggactggtccat |
| Dog-β-actin-mRNA-21 | aagcatttgcgatggacgat | PR8-NS-vRNA-21 | aactgacatgactcttgagg |
| Dog-β-actin-mRNA-22 | aaatgctacgcatctgctcg | PR8-NS-vRNA-22 | tctgtacctgcgtcgcgtta |
| Dog-β-actin-mRNA-23 | cttcggaattcactcatgca | PR8-NS-vRNA-23 | ggcacttaaaatgaccatgg |
| Dog-β-actin-mRNA-24 | catttgccagggccaattta | PR8-NS-vRNA-24 | gagcggattctgaaagaaga |
| Dog-β-actin-mRNA-25 | gcgcttattccagtttcatg | PR8-NS-vRNA-25 | acgtgctggaaagcagatag |
| Dog-β-actin-mRNA-26 | cagatacagcttcaaggaca | PR8-NS-vRNA-26 | tcggtctggacatcgagaca |
| Dog-β-actin-mRNA-27 | gttctacaatccatgctgat | PR8-NS-vRNA-27 | agaaatccctaagaggaagg |
| Dog-β-actin-mRNA-28 | tacaagtcaagatcagcaac | PR8-NS-vRNA-28 | tgatgccccattccttgatc |
| Dog-β-actin-mRNA-29 | ccaagggaacagttaacttg | PR8-NS-vRNA-29 | gagttgcagaccaagaacta |
| Dog-β-actin-mRNA-30 | cctggaataggcggtattaa | PR8-NS-vRNA-30 | tttctttggcatgtccgcaa |
| Dog-β-actin-mRNA-31 | acaactggttcagactcttg |  |  |
| Dog-β-actin-mRNA-32 | ttagaccggcaagacagaaa |  |  |
